# Deep learning-driven intelligent mesoscopic model (DeepMeso): a case study on ferroelectrics

**DOI:** 10.1093/nsr/nwag324

**Published:** 2026-05-28

**Authors:** Run-Lin Liu, Zhong-Hui Shen, Han-Xing Liu, Yang Shen, Ce-Wen Nan

**Affiliations:** State Key Laboratory of Advanced Technology for Materials Synthesis and Processing, Center of Smart Materials and Devices, Wuhan University of Technology, Wuhan 430070, China; School of Materials and Microelectronics, Wuhan University of Technology, Wuhan 430070, China; State Key Laboratory of Advanced Technology for Materials Synthesis and Processing, Center of Smart Materials and Devices, Wuhan University of Technology, Wuhan 430070, China; School of Materials and Microelectronics, Wuhan University of Technology, Wuhan 430070, China; State Key Laboratory of Advanced Technology for Materials Synthesis and Processing, Center of Smart Materials and Devices, Wuhan University of Technology, Wuhan 430070, China; School of Materials and Microelectronics, Wuhan University of Technology, Wuhan 430070, China; School of Materials Science and Engineering, State Key Laboratory of New Ceramics and Fine Processing, Tsinghua University, Beijing 100084, China; School of Materials Science and Engineering, State Key Laboratory of New Ceramics and Fine Processing, Tsinghua University, Beijing 100084, China

**Keywords:** deep learning, inverse design, mesoscopic models, latent diffusion model, ferroelectrics

## Abstract

Integrating artificial intelligence with computational methods has emerged as a transformative force in materials research, exemplified by breakthroughs such as DeepH and DeepMD. However, a critical gap persists in multiscale intelligent design: the lack of mesoscopic models capable of bridging microstructural features with macroscopic properties. Here, we develop DeepMeso, a generative deep learning-enabled mesoscopic model, for the intelligent design of complex heterogeneous materials. To exemplify the framework, we instantiate it in ferroelectrics (DeepFerro), where our workflow first integrates a data-driven surrogate model to predict key ferroelectric properties with an accuracy exceeding 99.6%. Then, we implement a 3D generative network to achieve inverse design across both composition and microstructure spaces, directly targeting predefined polarization objectives. When tasked with multi-objective on-demand generation, DeepFerro yields a mean squared error of 0.0497 and an *R*^2^ value of 95.44% against simulation benchmarks. Critically, it also exhibits robust transferability and extrapolation capability across 63 distinct ferroelectric systems, demonstrating its generalizability in end-to-end optimization. In parallel, model interpretability translates the learned relations into explicit, hierarchical guidelines. This generalizable intelligent design framework DeepMeso could be further extended to diverse heterogeneous materials, which will deepen the understanding of composition–microstructure–property relationships and facilitate the on-demand inverse design at the mesoscopic scale.

## Teaser

A generative mesoscopic intelligent design framework, DeepMeso, is constructed to connect composition, microstructure, and macroscopic properties, enabling accurate, transferable, and interpretable inverse design of complex heterogeneous materials.

## INTRODUCTION

Deep learning has been recognized as a promising approach that will revolutionize the role of computational simulations in materials research and development, including optimizing existing simulation workflows, enabling rapid performance prediction, and achieving precise intelligent design [1–4]. At the electronic/atomic scale, DeepH, a deep learning-based Hamiltonian prediction framework [5–8] provides a powerful Hamiltonian prediction framework for characterizing electronic states, while DeepMD, a deep learning-based molecular dynamics framework [9,10] facilitates large-scale molecular dynamics with near-density functional theory (DFT) accuracy. At the macroscale, Physics-informed Neural Networks [11,12] or operator learning [13,14] significantly accelerate partial differential equation solvers and enhance finite element simulation workflows through a synergistic approach combining data-driven and physical equations. Despite these advances, most approaches remain confined to either atomistic detail or continuum approximations. These isolated simulation scopes overlook the mesoscale, where the geometry and dynamics of mesoscopic microstructures serve as the natural bridge between microscopic mechanisms and macroscopic performance.

**Figure 1.**
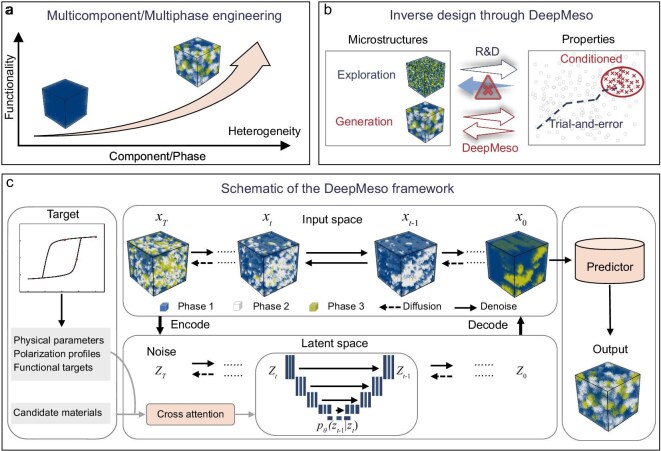
Overview of mesoscopic intelligent design model of DeepMeso. (a) The relationship between functionality design space and the heterogeneity in material multicomponent and multiphase engineering. (b) DeepMeso enables on-demand inverse design of microstructures. Traditional exploration relies on trial-and-error experiments and simulations to predict properties. In contrast, DeepMeso performs inverse generation of new microstructures tailored to diverse conditioned objectives. (c) Schematic of the DeepMeso framework presented in this work.

At the mesoscale, microstructural features such as phase distribution, defect evolution, and hierarchical structures significantly affect the macroscopic properties, as demonstrated across mechanical materials [15,16], structural materials [17–19], energy materials [20,21], and beyond. As shown in Fig. 1a, multicomponent and multiphase engineering at mesoscale expands the microstructural local heterogeneity and design flexibility by integrating the composition, distribution, morphology, and interface interaction of different components to achieve diverse functionalities [22–25]. However, current mesoscopic design relies on trial-and-error exploration through experimentation or physics-driven simulations such as phase-field simulations [26–28]. These methods can reveal microstructure–property relationships, but remain inherently oriented toward forward screening, requiring extensive iterations and computations across broad parameter spaces [29,30]. This bottleneck hinders rapid and directional inverse design.

Recent advances in generative deep learning models, such as variational autoencoders (VAEs) [31], generative adversarial networks [32,33], and diffusion models [34,35], have enabled on-demand inverse design by capturing intricate correlations between structures and properties from large-scale datasets. However, most studies focus primarily on the structural space itself, or on generation/reconstruction conditioned on simple and readily accessible scalar properties, with relatively limited efforts devoted to explicitly linking mesoscale microstructure generation to macroscopic properties. Here, we propose a framework that integrates generative deep learning with mesoscopic models (DeepMeso) to intelligently investigate the relationship between microstructure and macroscopic functional responses. Unlike many prevailing workflows that are still primarily limited to forward exploration of microstructure–property relationships, DeepMeso integrates high-accuracy forward prediction with inverse conditional generation, thereby enabling the design of candidate mesoscale ferroelectrics tailored to specific functional requirements (Fig. 1b). To demonstrate its applicability, we select ferroelectrics as a representative system because their tunable polarization–electric field (*P–E*) loops give rise to diverse functional requirements spanning energy storage, piezoelectric sensing, non-volatile memory, and related applications [36–39]. At the mesoscale, domain and phase engineering [40–46] expand the ferroelectric design space by tailoring microstructural configurations and interfacial interactions, thereby shaping the polarization switching energy landscape, the kinetics of domain walls, and macroscopic responses such as maximum polarization (*P*_max_), remanent polarization (*P*_r_), and coercive field (*E*_c_). However, the vast compositional and microstructural design space makes it highly challenging to efficiently identify and optimize multiphase configurations with targeted ferroelectric properties.

In this work, we demonstrate a ferroelectric mesoscopic intelligent design model (DeepFerro) as a ferroelectric-specific instantiation of the DeepMeso framework, that enables both forward prediction of candidate microstructures and inverse design of novel configurations across diverse material systems. First, a wide range of three-dimensional, multiphase microstructures are generated by emulating the nucleation and growth dynamics of mesoscale processes. Then, high-throughput phase-field simulations are used to establish a comprehensive composition–microstructure–property dataset. For forward design, a high-fidelity surrogate predictor is employed to map the spatiotemporal evolution of microstructures to their macroscopic responses with high accuracy. For inverse design, DeepFerro embeds the geometric complexity of multiphase microstructures into a pre-trained, informative low-dimensional latent space, where a latent diffusion model generates novel multiphase configurations conditioned on target ferroelectric responses (Fig. 1c). The strategy of classifier-free and predictor-guided screening was employed to generate new multiphase microstructures that are biased toward different property targets. Beyond scarce systems optimization, we demonstrate that DeepFerro generalizes across diverse material systems via transfer learning, exhibits extrapolative capability in functional scenarios constrained by limited data and provides hierarchical interpretability design guidelines. Together, these results highlight DeepMeso as a scalable paradigm for bridging composition–microstructure–property linkages and advancing inverse mesoscopic design across a wide range of complex materials.

## RESULTS

### Research workflow

Our method employed a predictor-guided generative model to conditionally generate ferroelectric multiphase microstructures. The model was conditioned on three distinct embeddings: (i) physical parameters (such as *P*_max_, *P*_r_, and *E*_c_), representing fundamental material characteristics; (ii) polarization profiles, encoded as sequence pairs of *P*–*E* loops; and (iii) functional targets, which define multiple desired performance metrics in specific application scenarios (Fig. 1c). In essence, the model learned a reversible mapping between ferroelectric properties and corresponding microstructures. First, a forward feature extraction framework was constructed, through which the latent representation of the microstructures was learned. A predictor was then created to accurately estimate the target property. Then, a latent diffusion model with classifier-free guidance [47] was used to iteratively denoise and sample the latent space. When combined with the predictor, the diffusion model could precisely produce ferroelectric microstructures that are biased toward the desired target properties. This framework allows for the sampling of the conditional distribution of arbitrarily broad and complex conditions. We also investigated the transferability of DeepFerro to previously unseen material systems by employing full fine-tuning. Even with limited training data, the model maintained high fidelity in polarization profile generation across different systems. Finally, using only 50 samples, we evaluated the extrapolation capability of DeepFerro for optimal functional targets in three representative application scenarios, demonstrating its capacity to efficiently extrapolate beyond the training domain and to explore the performance limits of ferroelectric microstructures.

### Forward prediction of ferroelectric properties via microstructures

**Figure 2.**
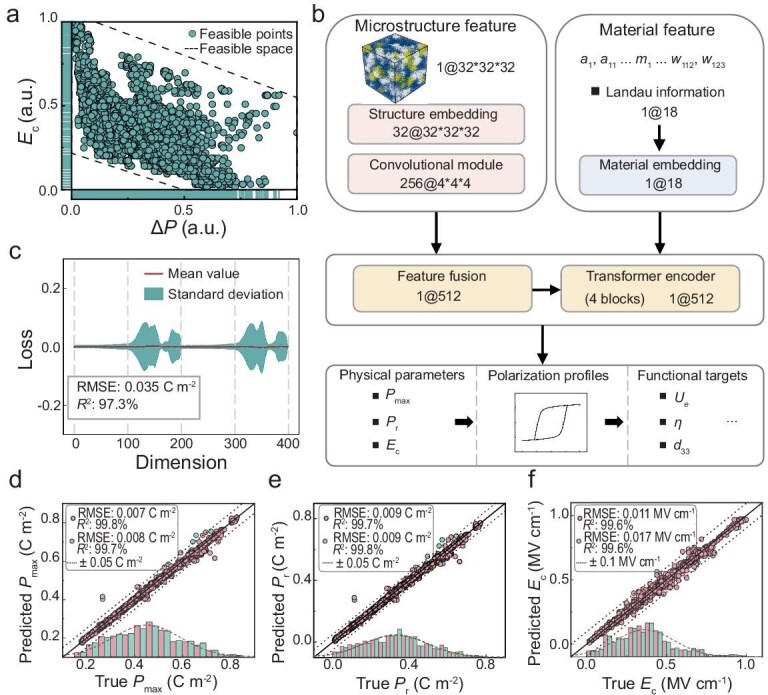
Dataset construction and surrogate predictor training. (a) Full design space of *E*_c_–Δ*P* for various ferroelectrics with different microstructures obtained by phase-field simulations. (b) Architecture of the predictor. Material and microstructural data are provided as input, and a representation of the microstructure is obtained through feature extraction and alignment. (c) Prediction loss in polarization profiles. Average MAE between predicted and ground-truth polarization values over 400 discrete electric field steps, each corresponding to a dimension in the output feature space. (d–f) Evaluations of physical parameters (*P*_max_, *P*_r_, *E*_c_) predictor model. The two point sets represent the training set with 4994 data points and the test set with 1249 data points, respectively.

To construct a comprehensive dataset for training DeepFerro, we designed a high-throughput algorithm for multiphase microstructure construction and employed phase-field simulations to capture the *P*–*E* responses of diverse ferroelectric systems. Here, the dataset was constructed using four representative ferroelectric materials of BaTiO_3_, PbTiO_3_, BiFeO_3_, and SrTiO_3_ as the foundational components. These materials span tetragonal, rhombohedral, and cubic crystallographic symmetries to enrich mesoscale accessible polarization space [41]. The resulting dataset consisted of three-dimensional microstructures represented by 32 × 32 × 32 voxels, incorporating single-, binary-, and ternary-phase mixtures. As detailed in Fig. S1, this resolution offers an optimal trade-off between computational accuracy and runtime, balancing model expressiveness with simulation efficiency. These microstructures were generated by capturing the fundamental nucleation and growth principles of materials, producing diverse structural types such as nanopolar clusters, lamellar, and nanopillar structures (see Note S1), underscoring their capacity to encapsulate the almost full spectrum of microstructure diversity experimentally observed in ferroelectric materials [48]. Phase-field simulations were used to calculate *P*–*E* responses for diverse microstructures under an applied electric field of 1 MV cm^−1^ (see ‘Methods’ section and Note S2). Thus, we constructed the composition–microstructure–property dataset comprising 6243 samples. This defines a dimensionless design space that spans a broad spectrum of non-linear *P–E* responses and representative ferroelectric behaviors. Figure 2a illustrates the correlation between the polarization difference (Δ*P = P*_max_ − *P*_r_) and *E*_c_, both of which are key parameters for characterizing *P*–*E* loop profiles. As shown, this dataset exhibits a wide variation range and covers a substantial portion of ferroelectricity space. Figure S2 demonstrates four distinct cases, illustrating how variations in material composition and microstructural configuration lead to pronounced differences in ferroelectric *P*–*E* behaviors.

Next, we developed a phase-field surrogate predictor to efficiently estimate the *P*–*E* loops with given microstructures, enabling rapid prediction and exploration of the design space. To improve model generalizability and prevent overfitting, we applied data augmentation via *z*-axis rotations and low-amplitude stochastic noise injection, while preserving the physical fidelity of the microstructures (see Note S3). This operation resulted in an expanded dataset of 19 929 composition–microstructure–property pairs. Subsequently, we trained a forward module using a three-dimensional convolutional neural network to predict polarization properties of a given microstructure as illustrated in Fig. 2b [49]. The module extracted spatial information from the microstructure through 3D convolution operations, and material characteristics were represented through a multilayer neural network embedding. These two types of information were then fused and input into a transformer layer for feature extraction and sequence information estimation (see Note S4). After applying a weighting method to account for sparse data during rapid domain flipping, the model achieved a mean *R*^2^ of 97.3% for polarization profiles comprising 400 sequential data points (Fig. 2c). As shown in Fig. 2d–f, all prediction accuracies of single physical parameters of *P*_max_, *P*_r_, and *E*_c_ exceed 99.6%. And an average difference of <0.1% between the test set and the training set indicates that the model is well generalized with no significant evidence of overfitting or underfitting observed. The forward module can effectively act as a surrogate model for complex theoretical calculations, completing the evaluation of polarization response under different applied electric fields in real time.

### Inverse design of microstructures for target properties

Although composition–microstructure–property relationships were well established for different ferroelectrics in the above modules, the inverse problem of designing materials with predetermined functionality lacks general solutions. Next, we focused on developing a demand-driven inverse design framework that is both accurate and scalable. After training the forward module, its parameters were frozen to serve as a fixed surrogate model, allowing the inverse module to screen ferroelectric microstructures on demand. We implemented a VAE with 3D residual blocks to compress high-dimensional microstructures into a low-dimensional latent space to enhance computational efficiency. This latent representation enabled a compact, information-rich domain by learning a low-dimensional manifold of the 3D microstructures. The encoder *E* mapped microstructures into the latent domain *z*, while the decoder *D* reconstructed *z* back into the microstructure data domain [50,51]. This framework explicitly decoupled the compression stage from the generative learning stage, requiring the autoencoder to be trained only once. The resulting latent space can be reused across multiple diffusion model trainings or adapted to entirely different downstream tasks (see Note S5). The voxel-based phase information was transformed into categorical features for training purposes, and the entire framework can be trained by minimizing the reconstruction error. As illustrated in Fig. S3, the confusion matrix shows a reconstruction accuracy of 99.9%. Consequently, microstructures can be represented by a low-dimensional latent space *z*, thereby ensuring the effective maintenance of their geometric features.

DeepFerro implemented a latent diffusion model with 3D U-Net convolutional neural network architecture for inverse microstructure generation. It supports two distinct conditioning strategies: physical parameters and polarization profiles (denoted as *y*). The model learned the conditional distribution *p*(*z*|*y*), where *z* represents the latent microstructure embedding, by iteratively denoising Gaussian noise. The latent U-Net backbone was enhanced with a cross-attention mechanism, enabling flexible conditional microstructure generation across different input modalities. To preprocess the various conditions *y*, a learnable embedding layer was introduced, which projects *y* into intermediate layers of the U-Net, allowing the model to adaptively focus on critical features (see ‘Methods’ section). Finally, the model jointly optimized the parameters *θ* of the conditional embedding layer and 3D U-Net through a loss function *L*_LDM_, ensuring coherent training of the encoder and generator for high-quality microstructure generation. Figure S4 visualizes the training process for both strategies, where the loss curves show stable convergence.

**Figure 3.**
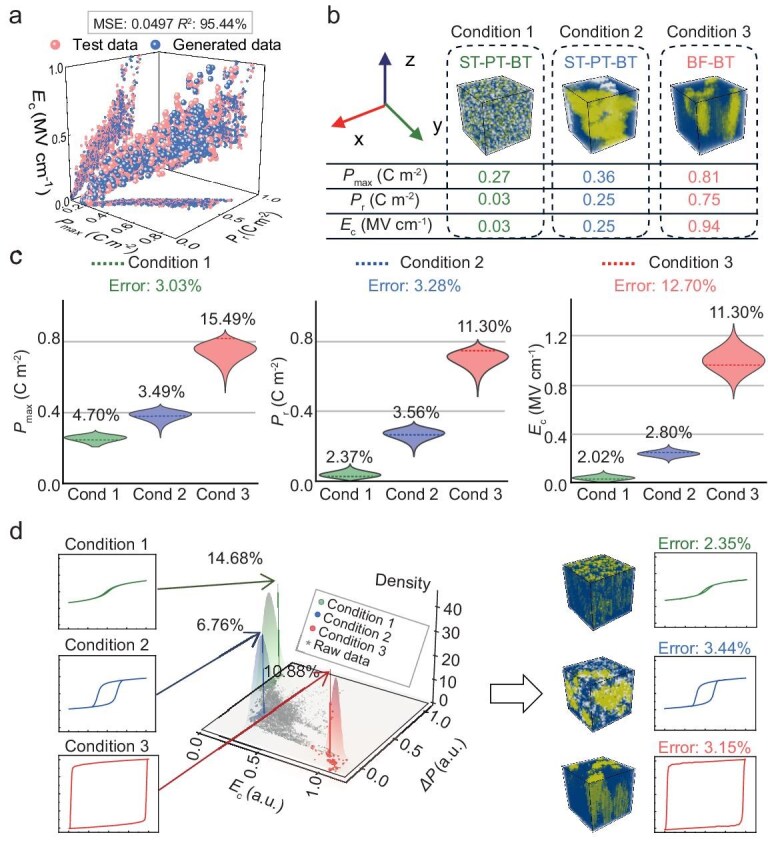
On-demand inverse design for target properties. (a) Comparisons of ferroelectric properties between test data and generated data for the task of multiple physical parameters generation. (b) Three typical conditions with corresponding microstructures and their *P*_max_, *P*_r_, and *E*_c_. (c) Violin plots of physical parameter distributions for different microstructures generated by DeepFerro. Dashed lines show target values under each condition, and values indicate the NRMSE relative to the target values. (d) The distribution between *E*_c_ and Δ*P* for microstructures generated under three polarization profiles. The dashed line indicates the target input polarization profile, and the height of each region represents the local properties density of the 100 microstructures generated by DeepFerro. Shown on the right are example microstructures obtained under each condition, together with their corresponding polarization profiles and the NRMSE.

While the training curves confirm stable convergence, the trade-off between quality and diversity during generation is also governed by the coefficient λ in classifier-free guidance [47]. We next investigated its role in multiparameter conditional generation, where the training objective was defined as the mean squared error (MSE) between the true and generated physical parameters. Table S1 summarizes the effect of λ on the generation performance in the test set. As λ increased, the model accuracy first increased and then decreased, reaching the lowest MSE (0.0497), the mean absolute error (MAE; 0.1497), and the highest *R*^2^ (95.44%) at λ = 3. The value was thus selected for subsequent modeling. Figure 3a compares the distribution of generated data with that of the original test set in a three-dimensional property space defined by three physical parameters. The generated samples exhibit strong overlap with the test data. Figure S5 further evaluates the generation performance of DeepFerro for each parameter, indicating good agreement between generated and true values of *P*_max_, *P*_r_, and *E*_c_ (*R*^2^ > 92%), with closely matched distributions. To further validate model performance, we evaluated DeepFerro using new representative targets. Figure 3b presents three generation conditions under different sets of physical parameters, each corresponding to a distinct microstructure. Using these conditions as targets, we generated 100 microstructures using DeepFerro and evaluated them with the predictor. Figure 3c presents the violin plots of parameter distributions for the generated microstructures. The normalized root mean square errors (NRMSEs) under the three conditions are 3.03%, 3.28%, and 12.70%, respectively. Slightly higher errors were observed under Condition 3, which correspond to the higher generation difficulties of extremely unique types of widest *P*–*E* loop. Nevertheless, the strong distributions in Fig. 3c still illustrate that DeepFerro reliably reproduces the multidimensional statistical characteristics of the materials, including both central tendencies and variability.

Based on the results with static physical parameters, we next investigated a more challenging task: generating microstructures conditioned on full polarization profile sequences. This task is considerably more demanding because the temporal polarization response is high-dimensional, non-linear, and highly sensitive to microstructural features. Based on the training results in Fig. S4, we further evaluated the model on the test set (1249 samples). The model achieved an MSE of 0.1884, an MAE of 0.2703, and an *R*^2^ of 84.58%. The broader evaluation range (−0.9 C m^−2^ to 0.9 C m^−2^ across all 400 electric field steps) and the complexity of evaluation profiles naturally lead to a modest reduction in accuracy, yet the model still captures the overall evolution of the polarization profiles. To examine this setting, we focused on three representative polarization profile conditions corresponding to typical *P*–*E* loop morphologies: slim (Condition 1), medium (Condition 2), and fat (Condition 3), as shown in Fig. 3d. This figure displays the three-dimensional density distributions of generated and true microstructure data corresponding to the three different *P*–*E* loop profiles. Dashed lines denote the target curves, while the colored point distributions reflect the statistics of the generated microstructures. The average NRMSE under the three polarization profile conditions is 14.68%, 6.76%, and 10.88% respectively, and the scattered points are closely arrange around the target lines. On the right side of Fig. 3d, three optimal microstructures generated under each condition, together with their corresponding polarization profiles, are presented. These newly generated structures differ from the originals (Fig. 3b) but also exhibit some similar trends, such as the comparable ferroelectric phase orientations in Condition 3. Their polarization profiles show the errors of 2.35%, 3.44%, and 3.15% relative to the inputs, suggesting that DeepFerro can effectively capture the overall curve morphology and subtle non-linear behaviors of realistic microstructures.

### Transferable design across different material systems

**Figure 4.**
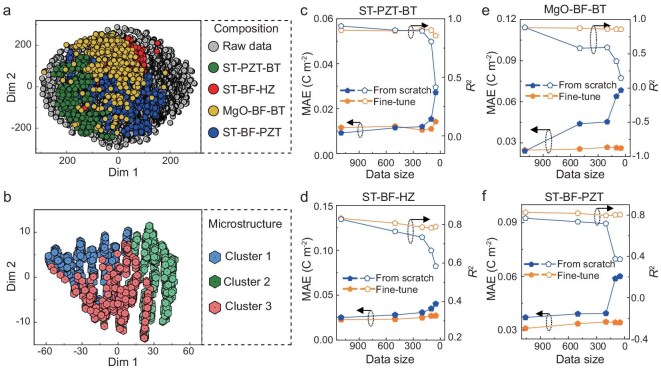
The transferability capability test of DeepFerro. (a) T-SNE visualization of polarization profile in the total raw dataset and the fine-tuned dataset, with colors representing different material systems. (b) T-SNE visualization of polarization profile of the MgO–BF–BT ternary system, with colors representing different structural clusters. (c–f) The variations of MAE and *R*^2^ with different data size during the transfer learning for different material systems of ST–PZT–BT, ST–BF–HZ, MgO–BF–BT, and ST–BF–PZT.

The forward and inverse design cycles can generate customized microstructures to meet user-specified objectives. However, the practical utility of the model depends on whether it can be effectively adapted to different material systems and remain robust under distribution shifts beyond those seen during pre-training. Therefore, achieving reliable design in unseen or new ferroelectric systems that differ in composition and microstructure is of greater practical importance. To address this issue, we applied transfer learning from the above version as a pre-trained model and adapted it to new material systems by updating the model parameters using limited data. Compared with training from scratch, full fine-tuning improves sample efficiency, accelerates optimization convergence, and increases training stability. Thus, we tested DeepFerro on a diverse set of previously unseen material systems, including both traditional ferroelectrics (e.g. Pb(Zr_0.52_Ti_0.48_)O_3_) [52] and emerging classes such as Hf-based (e.g. Hf_0.5_Zr_0.5_O_2_) [53] and typical simple oxide such as MgO [42,44]. Through ternary mixing, we considered 7 distinct material systems with 63 theoretically possible multicomponent combinations in the overall design space. We first performed phase-field simulations using an identical library of ∼1099 microstructures across 4 representative multiphase systems [SrTiO_3_–Pb(Zr_0.52_Ti_0.48_)O_3_–BaTiO_3_ (ST–PZT–BT), SrTiO_3_–BiFeO_3_–Hf_0.5_Zr_0.5_O_2_ (ST–BF–HZ), MgO–BiFeO_3_–BaTiO_3_ (MgO–BF–BT), and SrTiO_3_–BiFeO_3_–Pb(Zr_0.52_Ti_0.48_)O_3_ (ST–BF–PZT)] to obtain their *P*–*E* responses. As shown in Figs S7–S10, variations in phase fractions lead to pronounced changes in the polarization profiles for each system. We then analyzed the distribution of different material systems and microstructures in ferroelectric performance space to provide a direct visualization for transfer learning. Figure 4a shows a two-dimensional projection of hysteresis loops for four representative material systems, visualized using t-distributed stochastic neighbor embedding (t-SNE). Points with different colors represent distinct material systems, which remain within the raw property space but occupy different positions. This indicates significant differences in ferroelectric properties among different material systems, providing a basis for evaluating model transferability. Figure 4b presents a t-SNE visualization of polarization profiles for the MgO–BF–BT ternary system, where colors represent microstructural clusters identified by *k*-means clustering. Three clusters differ markedly in property space, with each showing characteristic microstructure–property correspondences (as shown in Fig. S11), suggesting that the pre-trained DeepFerro captures transferable microstructural features.

Then, we performed transfer learning across the four representative material systems using full fine-tuning to evaluate the ability of DeepFerro to generate microstructures on demand in previously unseen systems. During the training process, all model parameters were updated with no layers frozen, and the learning rate was reduced from 1 × 10^−3^ to 1 × 10^−4^ to ensure stable convergence and prevent overfitting. To accommodate the discrete nature of the material systems, one-hot encoding was applied. We then compared the performance of fine-tuned DeepFerro with the models trained from scratch under varying target dataset sizes with 1000, 500, 200, 100, and 50 samples. Here, 220 evaluations of inverse design tasks for on-demand polarization profiles were performed using respective test-set data as generation targets. As illustrated by the average MAE and *R*^2^ in Fig. 4c–f, fine-tuning consistently yields low errors (MAE < 0.04 C m^−2^) and high predictive fidelity (*R*^2^ > 0.78) across all four systems. Notably, when the training data are progressively reduced, the fine-tuned models remain robust: even with only 50 training samples, the decrease in *R*^2^ is <5%. In contrast, models trained from scratch deteriorate rapidly, exhibiting declines in *R*^2^ exceeding 29%. This indicates that DeepFerro, though not originally trained on these new material systems, can reliably generate ferroelectric microstructures on demand from limited data by leveraging the global features of *P*–*E* loops, thereby obviating the need for retraining.

### Extrapolation capability across diverse functional scenarios

Thus far, DeepFerro has enabled precise property prediction and on-demand inverse design, thereby empowering the exploration of theoretical property limits and discovering corresponding new multiphase microstructures. This capability becomes relevant in transferred material systems, where only limited fine-tuning data may be available and the accessible training samples often cover only a relatively narrow region of the property space. In this work, we used DeepFerro to probe the attainable property boundaries of the three-phase microstructure design space under various practical applications. To obtain as diverse ferroelectric responses as possible, we continued to select the above four representative multiphase systems for further exploration. Guided by application needs from the energy storage to non-volatile memory (see Note S7), we analyzed three pairs of key performance metrics: recoverable energy density (*U*_e_) versus efficiency (*η*); piezoelectric coefficient (*d*_33_) versus piezoelectric voltage coefficient (*g*_33_); and *E*_c_ versus *P*_r_. Their distributions (Fig. S12) reveal pronounced trade-offs that preclude simultaneous optimization, such as high *U*_e_ and *η*, high *d*_33_ and *g*_33_, or low *E*_c_ with high *P*_r_. Deciphering these inversely coupled performance parameters has long posed a major experimental challenge [54–56].

**Figure 5.**
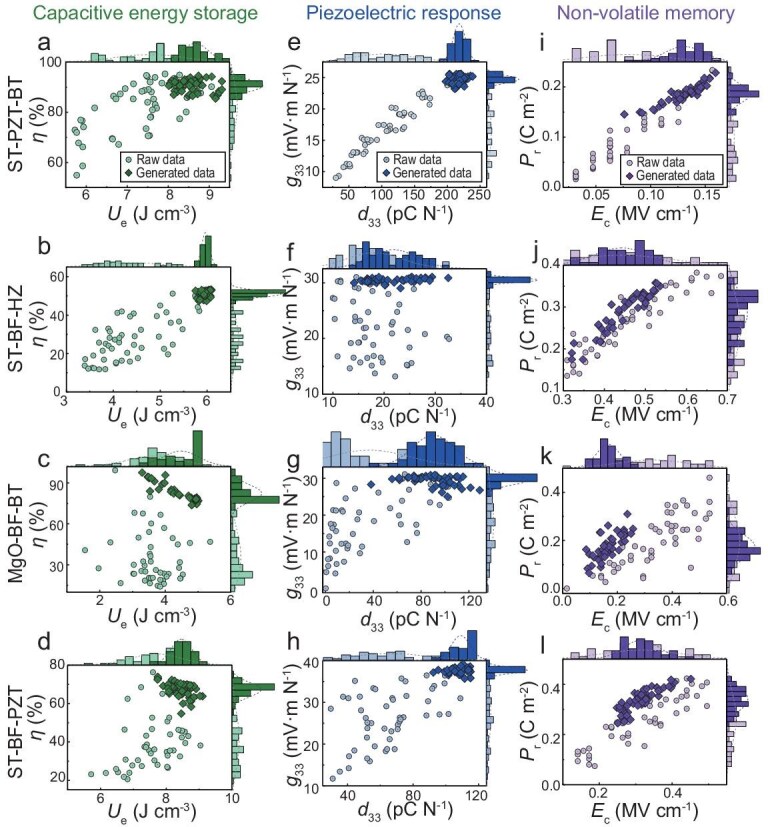
The extrapolation capability of DeepFerro in demand-driven generation tasks. Scatter plots with marginal distributions compare the raw dataset (open circles) and generated microstructures (solid diamonds) in three representative scenarios: (a–d) capacitive energy storage, characterized by recoverable energy density *U*_e_ and efficiency *η* (left); (e–h) piezoelectric response, characterized by piezoelectric coefficient *d*_33_ and piezoelectric voltage coefficient *g*_33_ (middle); and (i–l) non-volatile memory, characterized by coercive field *E*_c_ and remanent polarization *P*_r_ (right).

Here, we framed the inverse design of optimal microstructures for predefined functional targets as a dual-objective conditional generation task. For each application, a target vector y* = [*y*_1_*, *y*_2_*] was formed by pairing the best single-objective values observed in the raw data. The theoretical limit of a property is the best attainable value within microstructure space. During the adaptation process, we only modified the conditional encoding to accept functional targets. Then, DeepFerro was fine-tuned with only 50 random samples, and subsequently used to generate 50 new microstructures within the target spaces. Figure 5 shows the extrapolation capability of DeepFerro for four material systems in different application scenarios. In terms of capacitive energy storage (Fig. 5a–d), all generated microstructures exhibit both high energy densities and efficiencies (located at upper right with square dots), surpassing the distribution of the baseline of raw samples. Notably, for the ST–PZT–BT system, two generated microstructures delivered energy densities above 9 J cm^−3^ at 1 MV cm^−1^ and efficiencies over 90%, exceeding the best baseline samples and evidencing cross-system extrapolation. Figure S14 compares the 2D domain structures of the best raw microstructures with those of the optimal generated ones. The generated material systems exhibited more ordered multiphase patterns and regular domain configurations, which are theoretically conducive to higher recoverable energy density *U*_e_ and efficiency *η* [40,57,58]. Compared to conventional phase-field sampling, DeepFerro exhibited up to a 5-fold improvement in exploration efficiency, quantified as the fraction of microstructures with *U*_e_ and *η* simultaneously exceeding the 80th percentile of the raw dataset. For piezoelectric applications, the model extrapolated beyond the raw data distribution toward regions of higher *d*_33_ and *g*_33_ (Fig. 5e–h). In the ST–PZT–BT (Fig. 5e) and ST–BF–PZT (Fig. 5h) systems, the generated microstructures extend markedly into domains where both *d*_33_ and *g*_33_ were simultaneously enhanced. Notably, in the ST–PZT–BT system, generated microstructures exhibit *d*_33_ values that exceeded 200 pC N^−1^, with *g*_33_ exceeding 23 (mV m) N^−1^. In contrast, in systems with intrinsically weaker piezoelectric responses, such as ST–BF–HZ, extrapolation in *d*_33_ is very limited, and generated microstructures primarily shift toward higher *g*_33_. This phenomenon reflects that their raw data had already approached the property boundary, with *d*_33_ just restricted to 10–35 pC N^−1^. For non-volatile memory applications (Fig. 5i–l), DeepFerro achieves localized extrapolation beyond the raw dataset and surpassed the Pareto front of raw data. In the MgO–BF–BT system (Fig. 5k), the generated microstructures converge to the regions with lower *E*_c_ (<0.25 MV cm^−1^) and moderate *P*_r_, representing some improvements over the raw baseline. In other systems, owing to the strong positive correlation between *E*_c_ and *P*_r_ (Fig. S12c), the 50 raw samples readily encompass the property boundary, leaving little scope for pronounced extrapolation. Therefore, DeepFerro demonstrates a versatile and robust methodology to inversely search for optimal systems, efficiently probe theoretical property boundaries, and rapidly assess the potential of candidate materials for specific application scenarios.

### Hierarchical design guidelines for ferroelectrics

**Figure 6.**
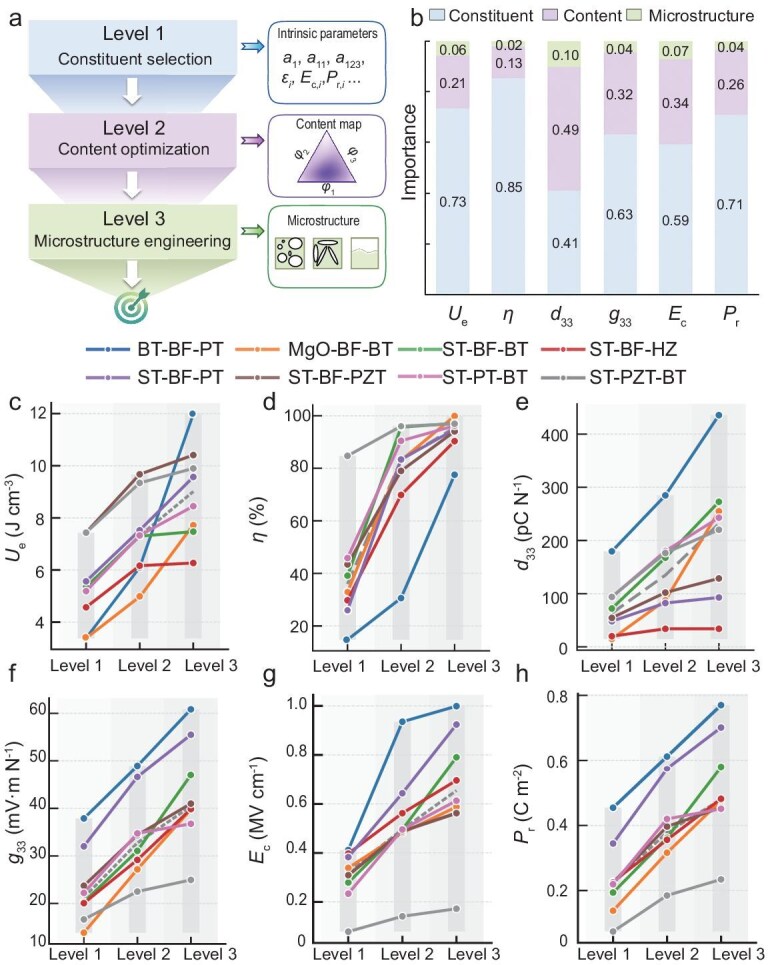
Hierarchical design guidelines for ferroelectrics. (a) Schematic workflow for the hierarchical design of multiphase ferroelectrics: constituent selection based on intrinsic parameters (level 1), content optimization with different combinations (level 2), and microstructure engineering through morphology, alignment, and interface control (level 3). (b) Feature importance aggregated over three descriptor families (constituent, content, and microstructure) for the six target performance metrics of *U*_e_, *η, d*_33_, *g*_33_, *E*_c_, and *P*_r_. (c–h) Performance trajectories of eight multiphase systems for *U*_e_, *η, d*_33_, *g*_33_, *E*_c_, and *P*_r_. Bars indicate the achievable design window spanned by all systems at each level, and lines with markers show the optimal response of each system.

While DeepFerro already enables high-accuracy and cross-system inverse design of multiphase ferroelectrics, a key question for experimentalists is how to translate such model predictions into a stepwise and interpretable design workflow starting from experimentally tunable variables. To this end, we formulated a three-level hierarchical optimization framework for ferroelectrics (Fig. 6a) based on multidimensional feature engineering. First, the constituent phases are chosen according to their intrinsic parameters. Then, we could optimize the phase volume fraction within the admissible content map. Finally, the mesoscale microstructure could be further refined to modulate the ferroelectric response. As shown in Table S3, 69 comprehensible descriptors are extracted and grouped into 3 families including constituent with some intrinsic phase parameters at level 1, phase volume fraction at level 2 and microstructure features with morphology, alignment and interfacial descriptors at level 3. To examine how descriptor groups are statistically associated with model prediction across different functional metrics, Fig. 6b compares the relative importance of the various descriptors across these metrics, as obtained from random forest regression models, which are used to characterize the statistical correlation between structure and performance. The predictive performance of the random forest models used for this analysis is summarized in Fig. S15, showing consistently good agreement between predicted and true values across the six target properties. The stacked bars show that constituent and content descriptors together account for the vast majority (>90%) of the total importance for all ferroelectric performance metrics, whereas microstructural descriptors provide a smaller but non-negligible contribution. This decomposition supports the general concept of this hierarchical design strategy: select appropriate constituent phases based on their intrinsic responses first, then design a suitable region of composition space, and finally tune the microstructure to refine the performance trade-offs.

To quantify how much performance could be changed as different design levels are activated, we carried out a three-level statistical analysis across all material systems and functional targets. For each system, we successively allowed design freedom within different levels, and recorded the representative observed performance attainable under the corresponding constraints. At level 1, for each system we defined the baseline as the mean performance computed over a subset of samples, in which all microstructural and content descriptors lay between the 10th and 90th percentiles of their overall distributions. At level 2, we only allowed volume fractions to vary freely and defined the achievable performance as the maximum values among all samples. At level 3, the upper bound was defined by the maximum observed performance in the full dataset. In Fig. 6c–h, the vertical bars show the observed performance envelope across all systems at each design level. To better show the magnitude and trend of the performance changes, we plotted the dashed lines to denote the median performance, and displayed colored curves to trace the level-by-level performance trajectories for individual systems. The accessible performance space shows a systematic expansion as each additional design level is introduced. For *U*_e_, for example, the median value increases from about 5.35 J cm^−3^ at level 1 to about 7.31 J cm^−3^ at level 2 (an increase of 36.5%), and further to about 9.01 J cm^−3^ at level 3 (a further gain of ∼23.2%). The median *η* rises from 39% to 83% between level 1 and level 2, and then to 95% after exploring all microstructure variation space at level 3. For *d*_33_, *g*_33_, *E*_c_, and *P*_r_, similar trends are observed: the medians typically increase by about 50% to 105% from level 1 to level 2, and from level 2 to level 3 microstructure engineering still delivers an additional improvement about 15% to 70%. These results demonstrate that constituent selection, content optimization, and microstructure engineering jointly constitute a hierarchical design framework, providing a practical and transferable pathway for targeted performance optimization in high-performance ferroelectrics. Therefore, our model enables on-demand inverse design, and provides an interpretable and executable hierarchical optimization strategy for experimental design.

## DISCUSSION

The rational design of mesoscopic heterogeneous materials faces inherent challenges due to their complex composition–structure–property relationships. To address this issue, we have proposed the DeepMeso framework at the mesoscopic scale and, as a case study, developed DeepFerro for ferroelectric materials. This framework enables both forward property prediction and on-demand inverse design, thereby effectively bridging multiscale modeling at the mesoscopic level. The principal advantage of DeepFerro lies in its ability to map local microstructures to macroscopic conditional properties, facilitating the rapid and precise generation of candidates that satisfy specific performance requirements. By leveraging transfer learning, the framework overcomes training-set limitations and extrapolates to new functional scenarios, accelerating the design of ferroelectric systems across application domains such as energy storage, sensing, and memory. With the continuous advances in diffusion model technology, such as Rectified Flow [59], Consistency Models [60], and Diffusion Transformer [61], it is expected that this framework will be extended to various complex mesoscopic material systems where sufficient data are available and both the microstructure and the target response can be reliably learned. Future extensions may also incorporate additional controllable variables, such as loading conditions, and leverage experimental data for calibration, thereby bringing this framework closer to practical experimental design. In summary, DeepFerro represents a successful instantiation of DeepMeso, an intelligent design model at the mesoscopic level, playing a key bridging role in guiding forward prediction and inverse design across multiple scales.

## METHODS

### *P*–*E* loop calculation using phase-field simulations

The order parameter *P* is selected to describe the time evolution of the polarization vector and domain structure. This is achieved by solving the three-dimensional time-varying Ginzburg–Landau equation in phase-field simulation:


(1) \begin{eqnarray*}
\frac{{\partial {P}_i(r,t)}}{{\partial t}} = - L\frac{{\delta {F}_{{\mathrm{total}}}}}{{\delta {P}_i(r,t)}}\,{\mathrm{ + }}\,{\xi }_i(r,t),(i = 1,2,3),
\end{eqnarray*}

where *L* is the kinetic coefficient related to the domain wall mobility, *F*_total_ is the total free energy of the system, *r* = (*x*_1_, *x*_2_, *x*_3_) represents the spatial vector, *t* denotes time, *P_i_* (*r, t*) represents the polarization component at a certain time and space position, and *ξ_i_* (*r, t*) is the impact of thermal noise, which is assumed to conform to a random Gaussian distribution.

### Theoretical basis of the diffusion process

By progressively denoising latent representations under target conditions, DeepMeso directly generates multiple candidate microstructures consistent with prescribed ferroelectric responses. The forward process proceeds in latent space, often defined as a Markov chain, where each step adds a certain amount of Gaussian noise to the data. In the initial stage, the voxel grid *x*_0_ is mapped to the latent space *z*_0_ = *E*(*x*_0_) by the encoder *E* [50]. At each diffusion step *t*, noise is added to the latent representation *z_t_*_−1_ to obtain *z_t_*:


(2) \begin{eqnarray*}
q({z}_t|{z}_{t - 1}) = {\mathrm{\mathcal{N}}}\left( {{z}_t;\sqrt {{\alpha }_t} {z}_{t - 1},{\beta }_tI} \right),
\end{eqnarray*}

where *β_t_* denotes the variance schedule at diffusion step *t*, which controls the amount of Gaussian noise added at each step. We further define *α_t_* = 1−*β_t_. N* represents the Gaussian distribution with mean $\sqrt {{\alpha }_{{t}}} {z}_{{{t}} - 1}$ and covariance *β*_t_*I*. By employing the Markov process, it is possible to ascertain the value of *x* corresponding to any given time step, beginning at *z*_0_. At each step of the forward process, noise is introduced.


(3) \begin{eqnarray*}
{z}_t &=& \sqrt {{\alpha }_t} {z}_{t - 1} + \sqrt {{\beta }_t} {\varepsilon }_t\\
&=& \sqrt {{\alpha }_t{\alpha }_{t - 1}} {z}_{t - 2} + \sqrt {1 - {\alpha }_t{\alpha }_{t - 1}} {\varepsilon }_{t - 1}\\
&=& \cdots \\
&=& \sqrt {{{\bar{\alpha }}}_t} {z}_0 + \sqrt {1 - {{\bar{\alpha }}}_t} \varepsilon ,
\end{eqnarray*}

where ${\varepsilon }_t \sim {\mathrm{\mathcal{N}}}(0,I)$, ${\bar{\alpha }}_t = \prod _{s = 1}^t{\alpha }_s$. A closed-loop distribution can be obtained, which shows that *z_t_* is a Gaussian perturbation of *z*_0_, with the mean being scaled and the variance increasing.


(4) \begin{eqnarray*}
q({z}_t|{z}_0) = {\mathrm{\mathcal{N}}}\left( {{z}_t;\sqrt {{{\bar{\alpha }}}_t} {z}_{t - 1},(1 - {{\bar{\alpha }}}_t)I} \right).
\end{eqnarray*}

In the reverse process, starting from a Gaussian noise sample *z_t_*, the model progressively denoises the latent representation to recover the original latent variable *z*_0_. To achieve this, the U-Net architecture is trained to learn the posterior noise distribution. The denoising step is formulated as


(5) \begin{eqnarray*}
{p}_\theta \boldsymbol{(}{z}_{t - 1}|{z}_t\boldsymbol{)} = {\mathrm{\mathcal{N}}}\left( {{z}_{t - 1};{\mu }_\theta \boldsymbol{(}{z}_t,t{\bf )},\sum {\boldsymbol{(}{z}_t,t\boldsymbol{)}} } \right).
\end{eqnarray*}

By iteratively applying this denoising procedure from *z_t_* to *z*_0_, the model reconstructs a structured latent representation, which is then decoded by *D*(*z*_0_) to generate the corresponding microstructure. After the generator produced candidate microstructures, the predictor evaluated the top-*k* results.

### Conditioning on polarization–electric field responses

The cross-attention mechanism is employed to integrate the conditional embedding data with the microstructure latent representation. During each denoising step, the cross-attention mechanism updated the latent representation according to the attention weights, thereby guiding the generation process.


(6) \begin{eqnarray*}
{\rm Attention} \left( {Q,K,V} \right)\ = {{soft}}\max \left( {\frac{{Q{K}^T}}{{\sqrt {{d}_k} }}} \right)V,
\end{eqnarray*}

where the Query (*Q*) represents the conditional embedding information, while the Key (*K*) and Value (*V*) correspond to the representations in the latent space. *d*_k_ denotes the dimensionality of the key, which is used for scaling the attention.

Classifier-free guidance is used to interpolate between unconditional and conditional model predictions to control the strength of conditioning during sampling. The adjusted noise estimate is defined as


(7) \begin{eqnarray*}
{\!\!\!\hat{\,\,\,\varepsilon }}_\Phi ({z}_t,c) = {\varepsilon }_\Phi ({z}_t,\emptyset ) + \lambda\! \cdot\! [{\varepsilon }_\Phi ({z}_t,c) - {\varepsilon }_\Phi ({z}_t,\emptyset )],
\end{eqnarray*}

where ${\varepsilon }_\Phi ({z}_t,c)$ denotes the predicted noise given condition *c*, and ${\varepsilon }_\Phi ({z}_t,\emptyset )$ corresponds to the unconditional prediction.

## Supplementary Material

nwag324_Supplemental_File

## Data Availability

All the codes used for this study are available from the corresponding authors upon request.
