# Supplementary material for: Deep learning-driven intelligent mesoscopic model (DeepMeso): a case study on ferroelectrics
Source: Natl Sci Rev. 2026 May 28;13(13):nwag324. doi: 10.1093/nsr/nwag324 (PMC13355323; doi:10.1093/nsr/nwag324)
Supplement: nwag324_Supplemental_File [file nwag324_supplemental_file.pdf]

Supplementary data for ‘Deep Learning-driven Intelligent Mesoscopic Model (DeepMeso): A case study on Ferroelectrics’

Contents

Supplementary Notes.....2

Supplementary Note 1 | Microstructure generation..... 2

Supplementary Note 2 | Phase-field simulations..... 3

Supplementary Note 3 | Data augmentation of the microstructures..... 6

Supplementary Note 4 | Predictor model..... 7

Supplementary Note 5 | The details of VAE for latent diffusion..... 8

Supplementary Note 6 | 3D U-Net architecture..... 9

Supplementary Note 7 | Quantitative evaluation of metrics from *P–E* loops across diverse functional scenarios...... 11

Supplementary Figures..... 13

Supplementary Figure 1 | The trade-offs between computational accuracy and runtime for different grid points..... 13

Supplementary Figure 2 | The different *P–E* loops achievable through structural and compositional variation..... 14

Supplementary Figure 3 | The confusion matrix in the VAE model on microstructures. All diffusion processes are based on VAE of a perceptual compression model..... 15

Supplementary Figure 4 | Evolution of training and test loss over training epochs..... 16

Supplementary Figure 5 | The generation performance of DeepFerro based on 3 physical parameter targets (P_{max}, P_r and E_c) on the test set..... 17

Supplementary Figure 6 | Examples of microstructure generation evaluation with polarization profile conditions for two typical ferroelectric systems..... 18

Supplementary Figure 7 | *P-E* hysteresis loops of ST-BF-PZT multiphase solid solutions using phase-field simulation..... 19

Supplementary Figure 8 | *P-E* hysteresis loops of ST-BF-HZ multiphase solid solutions using phase-field simulation..... 20

Supplementary Figure 9 | *P-E* hysteresis loops of ST-PZT-BT multiphase solid solutions using phase-field simulation...... 21

Supplementary Figure 10 | *P-E* hysteresis loops of MgO-BF-BT multiphase solid solutions using phase-field simulation. 22

Supplementary Figure 11 | Observed correlations between structural clusters and ferroelectric polarization behavior 23

Supplementary Figure 12 | Distributions of functional performance targets in capacitive energy storage, piezoelectric sensing and non-volatile memory scenarios 24

Supplementary Figure 13 | Performance evaluation of the predictor in capacitive energy storage and piezoelectric sensing applications in test set 25

Supplementary Figure 14 | 2D domain structure corresponds to Fig. 5a........ 26

Supplementary Figure 15 | Predictive performance of the random forest models used for feature importance analysis. 27

Supplementary Tables 28

Supplementary Table 1 | The effect of Guidance scale λ on the generation performance. 28

Supplementary Table 2 | Comparison of methods, software and running speeds for different tasks in materials microstructure design........ 29

Supplementary Table 3 | Definitions of the constituent, content and microstructural descriptors forming the hierarchical design space. 30

References 32

Supplementary Notes

Supplementary Note 1 | Microstructure generation

This algorithm is used to generate the three-dimensional microstructure of multiphase ferroelectrics, consistent with domain patterns observed experimentally through imaging and characterization techniques. The process begins by creating an empty three-dimensional matrix, followed by random growth of phase nuclei and extensions according to the specified volume fractions, until the target microstructure is formed. This process can be completed in the following steps[1]: Firstly, a three-dimensional matrix M with dimensions (i_{max}, j_{max}, k_{max}) is generated. The initial value of each position in the matrix is 0, representing the matrix phase. The coordinates (i, j, k) of the

matrix represent the points in space. For each position in the matrix, a uniformly distributed random number $r_{ijk} \in [0,1]$ is generated. According to the input volume fraction probability P_{growth} of the first or second phase, if the random number r_{ijk} is less than P_{growth}, the position becomes a growth nucleus and $M(i, j, k)$ is set to 1 or 2. For each growth nucleus, the 26 neighborhoods around it are traversed (including six directions such as up, down, left, right, front and back and their neighboring points, a total of 26 directions). Starting from each nucleus, a number $r_{growth} \in [0,1]$ is randomly generated, and according to the corresponding growth probability P_{growth}, it is determined whether to grow in that direction.

$$M(i, j, k) = \begin{cases} \text{phase}_n, & \textit{if} \ \ r_{growth} < P_{growth}, \\ 0, & \text{otherwise.} \end{cases} \tag{S1}$$

$M(i, j, k)$ is an adjacent point that is set to 1 or 2, marking the point as the corresponding phase. This process is performed recursively until the nuclei expand to reach the target volume fraction. To ensure the diversity and representativeness of the microstructures, over 1000 distinct microstructure configurations were constructed, covering a broad spectrum of morphologies (such as vertical, horizontal, lamellar, granular, layered, etc.).

Supplementary Note 2 | Phase-field simulations

In general, the total free energy F_{total} can be expressed as the integral of the free energy density, which is composed of the Landau bulk free energy (f_{bulk}), gradient energy (f_{grad}), elastic energy (f_{elas}), and electric energy (f_{elec}). The total energy can be expressed as follows:

$$F_{total} = \int_V [\, f_{bulk} + f_{grad} + f_{elas} + f_{elec}] dV, \tag{S2}$$

where V is the volume of the system. In terms of polarization, the f_{bulk} of an stress-free ferroelectric can be expressed as a sixth-order expansion as follows:

$$
\begin{aligned}
f_{\text{bulk}} &= a_1(P_1^2+P_2^2+P_3^2)+a_{11}(P_1^4+P_2^4+P_3^4) \\
&+a_{12}(P_1^2P_2^2+P_2^2P_3^2+P_1^2P_3^2)+a_{111}(P_1^6+P_2^6+P_3^6) \\
&+a_{112}\left[P_1^4(P_2^2+P_3^2)+P_2^4(P_1^2+P_3^2)+P_3^4(P_1^2+P_2^2)\right]+a_{123}P_1^2P_2^2P_3^2,
\end{aligned} \tag{S3}
$$

where $a_1, a_{11}, a_{12}, a_{111}, a_{112}, a_{123}$ are Landau coefficients, which is considered locally inhomogeneous. Owing to the contribution of domain walls, the gradient energy f_{grad} is expressed as follows:

$$
\begin{aligned}
f_{\text{grad}} &= \frac{1}{2}G_{11}(P_{1,1}^2+P_{2,2}^2+P_{3,3}^2)+G_{12}(P_{1,1}P_{2,2}+P_{2,2}P_{3,3}+P_{1,1}P_{3,3}) \\
&+\frac{1}{2}G_{44}\left[(P_{1,2}+P_{2,1})^2+(P_{2,3}+P_{3,2})^2+(P_{1,3}+P_{3,1})^2\right] \\
&+\frac{1}{2}G'_{44}\left[(P_{1,2}-P_{2,1})^2+(P_{2,3}-P_{3,2})^2+(P_{1,3}-P_{3,1})^2\right],
\end{aligned} \tag{S4}
$$

where G_{ij} is gradient energy coefficient, and $P_{i,j}=\frac{\partial P_i}{\partial x_j}$. The elastic energy density can be expressed as:

$$
f_{\text{elas}} = \frac{1}{2}c_{ijkl}e_{ij}e_{kl} = \frac{1}{2}c_{ijkl}(\varepsilon_{ij}-\varepsilon_{ij}^0)(\varepsilon_{kl}-\varepsilon_{kl}^0), \tag{S5}
$$

where c_{ijkl} is the elastic stiffness tensor, e_{ij} represents the elastic strain, ε_{ij} represents the total elastic strain, ε_{ij}^0 is the eigenstrain expressed as $\varepsilon_{ij}^0 = Q_{ijkl}P_kP_l$, and Q_{ijkl} is the electrostrictive coefficient. The electrostatic energy density f_{elec} of the system is composed of an external electric field and depolarization field.

$$
f_{\text{elec}} = -P_i(r)E_i(r)-\frac{1}{2}\varepsilon_0\kappa_{ij}^bE_i(r)E_j(r), \tag{S6}
$$

where $\kappa_{ij}^b(r)$ is the background dielectric constant tensor.

The model size in three-dimensional simulation is 32×32×32 grid points, grid distance is Δx=Δy=Δz=1.0 nm, and periodic boundary conditions are adopted. Comparisons are drawn by the trade-offs between computational accuracy and runtime for different grid points. To balance accuracy and computational cost, we define a composite score (*S*):

$$S_i = \frac{1-NE_i}{\log(1+T_i)}, \tag{S7}$$

$$NE_i = \frac{MSE_i\text{-}MSE_{ref}}{MSE_{max}\text{-}MSE_{ref}}, \tag{S8}$$

where NE_i is the normalized mean squared error of at the i-th grid level relative to the baseline (128-grid), and T_i denotes corresponding runtime (in minutes). A grid size of 32 achieves a favorable trade-off between accuracy and computational time.

In this work, the voltage was applied along [001] direction under a unified loading protocol, up to 1 MV cm^{-1}, under which all samples can drive stable domain switching and produce complete *P–E* loops, thereby preserving both robust feature extraction and discriminability among different microstructures and material systems. For each sample, the *P–E* response was represented as a sequence of 400 points sampled along a predefined sinusoidal electric-field loading path. The same field trajectory and sampling density were used for all samples throughout data generation and model training. And the temperature is 300 K. The calculation is based on the relevant parameters of previous studies as follows:

For ferroelectric phase, five set of landau coefficients ($BaTiO_3$, $PbTiO_3$, $Pb(Zr_{0.52}Ti_{0.48})O_3$, $Hf_{0.5}Zr_{0.5}O_3$ and $BiFeO_3$) are used respectively[2–5],

a_1 = 3.34×10^5(T-381) m^2·N C^{-2}·, a_{11} = 4.69×10^6(T-393)-2.02×10^8 m^6·N C^{-4}, a_{12} = 3.23×10^8 m^6·N C^{-4}, a_{111} = -5.52×10^7(T-393)+2.76×10^9 m^{10}·N C^{-6}·, a_{112} = 4.47×10^9 m^{10}·N C^{-6}, a_{123} = 4.91×10^9 m^{10}·N C^{-6};

a_1 = 3.8×10^5(T-752.15) m^2·N C^{-2}, a_{11} = -7.3×10^7 m^6·N C^{-4}, a_{12} = 7.5×10^8 m^6·N C^{-4}, a_{111} = 2.6×10^8 m^{10}·N C^{-6}, a_{112} = 6.1×10^8 m^{10}·N C^{-6}, a_{123} = -3.7×10^9 m^{10}·N C^{-6};

a_1 = 1.45×10^5(T-387.06) m^2·N C^{-2}, a_{11} = 5.83×10^7 m^6·N C^{-4}, a_{12} = 1.82×10^8 m^6·N C^{-4}, a_{111} = 1.5×10^8 m^{10}·N C^{-6}, a_{112} = 6.88×10^8 m^{10}·N C^{-6}, a_{123} = -3.24×10^9 m^{10}·N C^{-6};

a_1 = -4×10^8 m^2·N C^{-2}, a_{11} = 3.7×10^9 m^6·N C^{-4}, a_{12} = 0 m^6·N C^{-4}, a_{111} = 1.1×10^9 m^{10} N C^{-6}, a_{112} = 0 m^{10}·N/C^6, a_{123} = 0 m^{10}·N C^{-6};

a_1 = 4.64385×10^5(T-1103) m^2·N C^{-2}, a_{11} = 2.29×10^8 m^6·N C^{-4}, a_{12} = 3.0636×10^8 m^6·N C^{-4}, a_{111} = 5.9919×10^7 m^{10}·N C^{-6}, a_{112} = -3.3398×10^5 m^{10}·N C^{-6}, a_{123} = -1.7775×10^8 m^{10}·N C^{-6}.

For paraelectric cubic phase, one set of landau coefficients ($SrTiO_3$) is used[6],

a_1 = 7.06×10^5(T-36.5) m^2·N C^{-2}, a_{11} = 1.7×10^9 m^6·N C^{-4}, a_{12} = 1.37×10^9 m^6·N C^{-4}, a_{111} = a_{112} = a_{123} = 0 m^{10}·N C^{-6}.

For linear phase, MgO is considered which landau coefficients are as follow[7],

a_1 = 5.65×10^9 m^2·N C^{-2}, a_{11} = a_{12} = 0 m^6·N C^{-4}, a_{111} = a_{112} = a_{123} =0 m^{10}·N C^{-6}.

The gradient energy coefficient, elastic coefficient and electrostrictive coefficient of the entire system are as follows:

G_{11}/G_{110} = 1.2, G_{12}/G_{110} = 0, G_{44}/G_{110} = G'_{44}/G_{110} = 0.6, G_{110} = 7.04×10^{-11} m^4·N C^{-2}·; C_{11} = 3.2×10^{11} N m^{-2}, C_{12} = 1.1×10^{11} N m^{-2}, C_{44} = 1.3×10^{11} N m^{-2}; Q_{11} = 6.6×10^{-2} m^4 C^{-2}, Q_{12} = -1.35×10^{-2} m^4 C^{-2}, Q_{44} = 4.8×10^{-3} m^4 C^{-2}.

Supplementary Note 3 | Data augmentation of the microstructures

To increase the diversity of the data and consider the prediction fluctuations, we performed data augmentation on each of these microstructures. These methods expanded the range of variation in the training set through rotation and symmetry operations while ensuring that the essential characteristics of the microstructures were not changed under the premise of physical consistency. The specific operations are as follows:

$$F(i,j,k) = F\left(R_z(i,j,k) + \varsigma\right), \quad \text{(S9)}$$

where $R_z(i,j,k)$ represents the rotation around the z-axis, which randomly selects a rotation angle of 90°, 180°, 270° or 360° to generate microstructures with different spatial orientations, and ς represents random Gaussian noise to simulate interferences and random errors that may occur during the manufacturing process or use of materials.

Supplementary Note 4 | Predictor model

To achieve rapid and accurate prediction of ferroelectric microstructure properties, we developed a deep learning-based surrogate model capable of processing three-dimensional voxel-based microstructure data. The model architecture is implemented in Python 3.8, comprising three key components:

1. Structure Embedding: The input 3D microstructure data $X \in \mathbb{R}^{N \times H \times W \times D}$ are processed by convolutional layers to extract multi-scale spatial features:

$$F = \mathrm{Conv}_{3D}(X) + \mathrm{DilatedConv}_{3D}(X), \tag{S10}$$

where H, W, D are the spatial dimensions, and N is the number of microstructures. Residual connections are applied to preserve information flow.

2. Feature Fusion: Extracted spatial features F are integrated with material descriptors M through a fully connected layer:

$$Z = \sigma(\mathrm{W}_1 F + \mathrm{W}_2 M + \mathrm{b}), \tag{S11}$$

An optional Transformer Encoder further refines Z to capture long-range dependencies, particularly polarization dynamics near coercive fields.

3. **Optimized Training:** A weighted loss function is introduced to handle data imbalance, particularly the scarcity of domain-switching data:

$$\zeta = \sum_{i=1}^{N} \omega_i \cdot \ell(\hat{y}_i, y_i), \tag{S12}$$

where ω_i is the weight of each sample, ℓ is the loss function, and N is the number of samples. The model is trained using an 80/20 train-test split and the Adam optimizer with an adaptive learning rate (ReduceLROnPlateau) to ensure convergence.

For *P*–*E* loop profiles prediction, a weighting method was used to increase the weight of sparse data in the ferroelectric domain flipping process based on the standard deviation of each dimension. Specifically, the weight τ_i of each sampling point *i* is written as:

$$\tau_i = 1 + \gamma \frac{|P_i - P_{i-1}|}{\max_{\mathrm{j}} |P_i - P_{i-1}| + \delta} \tag{S13}$$

Where $|P_i - P_{i-1}|$ represents the magnitude of the local polarization change, γ is a hyperparameter controlling the weighting intensity, and δ is a numerical stability term.

Supplementary Note 5 | The details of VAE for latent diffusion

To enhance the efficacy of structural generation and more effectively address intricate microstructures, DeepFerro employs an independent VAE model to transform sparse voxel-based microstructures into a low-dimensional latent space. In this latent space, the data distribution is more straightforward and uniform, which facilitates more efficient processing by the diffusion model. In comparison to previous complex methods that necessitate the learning of encoder/decoder architectures and score-based priors simultaneously, this method no longer entails the need for intricate trade-offs between reconstruction and generation capabilities. This ensures the attainment of exceptionally high reconstruction accuracy while requiring minimal regularization of the latent space[8]. In particular, when a voxel space $x \in \mathbb{R}^{L \times W \times H \times 3}$ is provided, the encoder *E* encodes it into a latent representation $z = E(x)$, and the decoder *D*

reconstructs the microstructure x from the latent representation, resulting in $\tilde{z} = D(z) = D(E(x))$ where $z \in \mathbb{R}^{l \times w \times h \times c}$. The downsampling factor $f = H/h = W/w$ was set to 4, thereby establishing a suitable compromise between computational efficiency and accuracy. The specific loss function is as follows:

$$L_{recon} = -\sum_{i}[x_i \log(\hat{x}_i) + (1 - x_i)\log(1 - \hat{x}_i)], \tag{S14}$$

$$L_{KL} = D_{KL}(q(z \mid x) \parallel p(z)) = \frac{1}{2}\sum_{j}(1 + \log(\sigma_j^2) - \mu_j^2 - \sigma_j^2), \tag{S15}$$

$$L = L_{\mathrm{recon}} + m * L_{reg}, \tag{S16}$$

where L_{recon} represents the reconstruction error, which is calculated using cross-entropy loss. L_{KL} represents the regularization term, expressed as *KL* divergence, which is employed to quantify the discrepancy between the learned latent distribution and the standard Gaussian distribution. This penalty term compels the latent space distribution of the encoder output to gravitate towards the standard normal distribution, thus maintaining the structural integrity of the latent space in a coherent and systematic manner. The parameter *m* serves to regulate the relative significance of the reconstruction error and the regularization term. In this training process, *m* is set to 0.01.

Supplementary Note 6 | 3D U-Net architecture

As the forward process is fixed, the training process allows encoder *E* to effectively obtain z_t. The samples of p(z) can then be decoded into the microstructure space by decoder *D*. The neural backbone is implemented as a time-conditioned 3D U-Net, which performs noise prediction and step-by-step denoising by changing the network architecture. The optimization goal $L_{\mathrm{DeepFerro}}$ is:

$$L_{\text{DeepFerro}} = \mathbb{E}_{z,\varphi\sim N(0,1),t}[\| \varphi - \varphi_\theta(z_t, t) \|_2^2]. \tag{S17}$$

The 3D U-Net architecture progressively reduces spatial information while increasing latent feature representation, thereby reducing the reliance on spatial domain latent representations. In particular, the 3D U-Net integrates CNN, residual networks, and spatial self-attention mechanisms to facilitate the efficient processing of sparse 3D data and enable precise reconstruction. The input data is initially mapped to 160 channels through a convolutional layer, with time embeddings employed to capture temporal information from the diffusion model. During the downsampling stage, multiple DownBlock3D and ResNet3D modules extract high-dimensional features in a progressive manner, while S-shaped activation functions and spatial self-attention mechanisms capture long-range spatial dependencies, thereby reducing computational complexity. The intermediate stage comprises two ResNet3D modules and one Transformer3D module. The latter employs eight attention heads (with a dimensionality of 32 per head) to effectively enhance the model's capacity for learning long-range dependencies. In the upsampling stage, the UpBlock3D modules gradually restore the spatial resolution, with cross-layer connections fusing features to ensure accurate detail reconstruction.

By progressively denoising latent representations under target conditions, DeepMeso directly generates multiple candidate microstructures consistent with prescribed ferroelectric responses, rather than iteratively updating candidates through search operations such as optimization-based generation methods. The diffusion process is trained with a PNDM scheduler using 1000 diffusion timesteps. The variance schedule β_t follows a scaled linear schedule, increasing monotonically from 0.00085 to 0.012. This schedule progressively transforms the latent representation from structured signal to near-Gaussian noise in the forward process, and correspondingly determines the denoising trajectory during inverse sampling.

Supplementary Note 7 | Quantitative evaluation of metrics from *P–E* loops across diverse functional scenarios.

Ferroelectric materials support a broad range of functional applications, and each associated with distinct quantitative evaluation derived from their hysteresis behavior.

1. **Capacitive energy storage:** For capacitive energy storage, the recoverable energy density U_e and efficiency η are calculated from the area enclosed between the charge and discharge segments of the *P–E* loop, specifically[9]:

$$U_{total} = \int_0^{P_{max}} E(P)dP, \tag{S18}$$

$$U_e = \int_{P_r}^{P_{max}} E(P)dP, \tag{S19}$$

$$\eta = \frac{U_e}{U_{total}}. \tag{S20}$$

These equations demonstrate that high P_{max}, low P_r and high dielectric breakdown field E_b are conducive to achieving higher energy density and energy efficiency in dielectric materials.

2. **Piezoelectric response:** In piezoelectric materials, the effective longitudinal piezoelectric coefficient d_{33} arises from the coupling between spontaneous polarization and electrostrictive strain. It can be approximated by[10]:

$$d_{33} \approx 2Q_{33} \cdot \varepsilon \cdot P_s, \tag{S21}$$

where Q_{33} is the electrostrictive coefficient, the dielectric permittivity ε is calculated using effective medium theory, and the spontaneous polarization P_s is extracted as the intercept of the tangent to the *P-E* loop at the point of maximum polarization. The piezoelectric voltage coefficient g_{33} quantifies the electric field generated per unit mechanical stress along the polarization axis, which defined as:

$$g_{33} = \frac{d_{33}}{\varepsilon}. \tag{S22}$$

Achieving high d_{33} together with high g_{33} is vital for maximizing electromechanical conversion efficiency and enabling multifunctional device applications.

3. **Ferroelectric non-volatile memory:** For non-volatile ferroelectric memory, desirable characteristics include remanent polarization P_r and low coercive field E_c. A higher P_r ensures better retention and stability, while a lower E_c indicates lower power consumption during switching[11].

Supplementary Figures

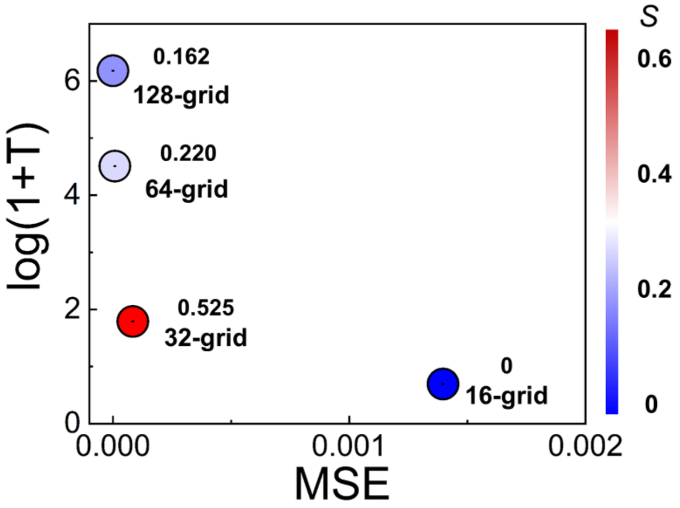

Supplementary Figure 1. The trade-offs between computational accuracy and runtime for different grid points. The color represents the magnitude of the composite score *S* at different grid size.

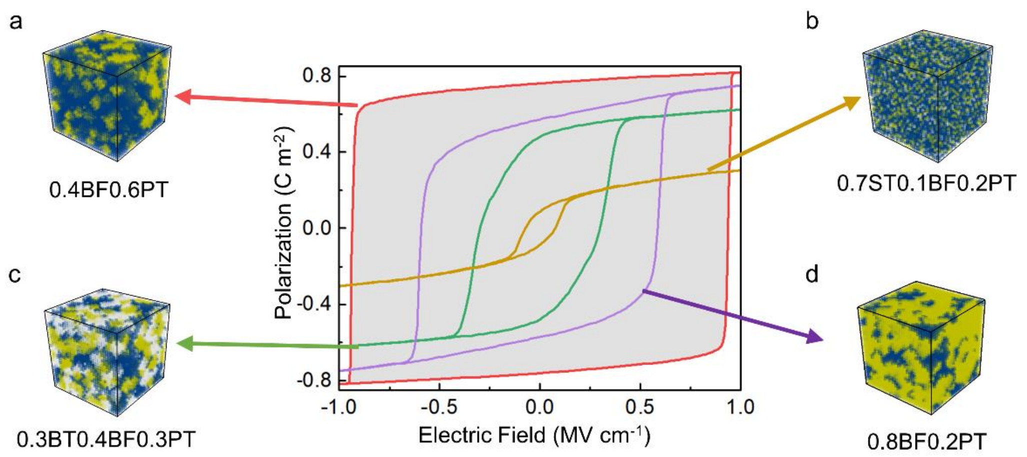

Supplementary Figure 2. The different *P–E* loops achievable through structural and compositional variation. (a) A composite of ferroelectric $BiFeO_3$ and $PbTiO_3$ (0.4:0.6) exhibiting a saturated *P–E* loop. (b) A ternary composition of paraelectric $SrTiO_3$, $BiFeO_3$, and $PbTiO_3$ (0.7:0.1:0.2), showing a highly disordered and slender *P–E* loop. (c) A ternary composition of $BaTiO_3$, $BiFeO_3$, and $PbTiO_3$ (0.3:0.4:0.3). (d) A binary system of $BiFeO_3$ and $PbTiO_3$ (0.8:0.2).

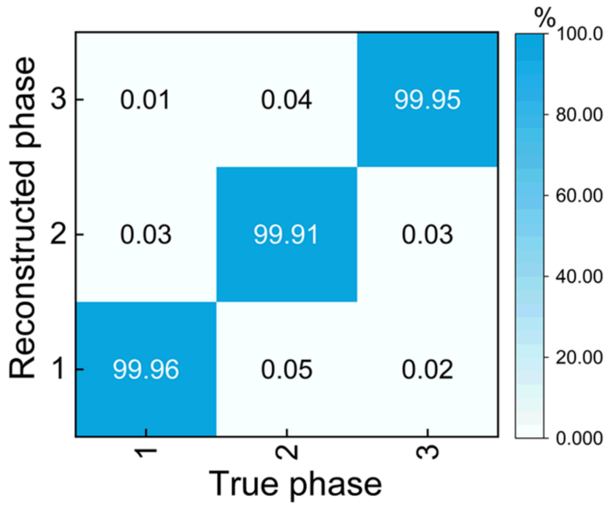

Supplementary Figure 3. The confusion matrix in the VAE model on microstructures. All diffusion processes are based on VAE of a perceptual compression model. It evaluates the correspondence between the true phase categories and the reconstructed phase categories. The confusion matrix is presented in percentage format, illustrating the prediction distribution for each class. Rows represent the true phase categories, while columns indicate the phase categories reconstructed by the model.

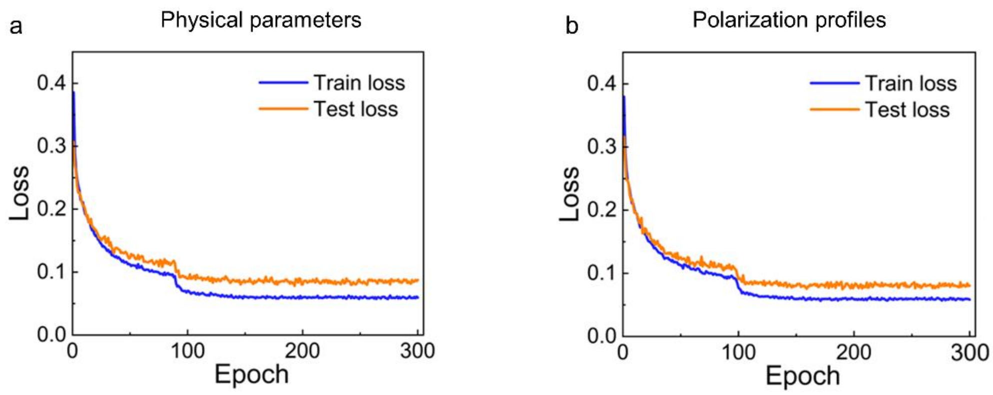

Supplementary Figure 4. **Evolution of training and test loss over training epochs.** (a) Model trained on multiple physical parameters. (b) Model trained on high-dimensional polarization profiles. In both cases, training and test losses decrease rapidly during the initial epochs, followed by a slower convergence and stabilization after approximately 100 epochs.

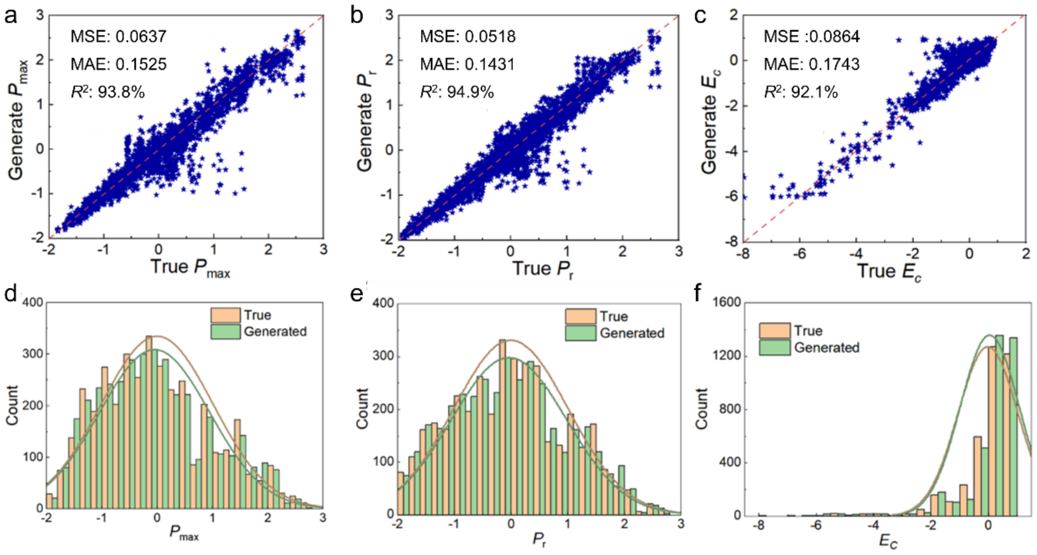

Supplementary Figure 5. The generation performance of DeepFerro based on 3 physical parameter targets (P_{max}, P_r and E_c) on the test set. (a–c) Scatter plots of generated versus true values for key physical parameters that are normalized. All three targets exhibit strong agreement, with R^2 scores above 92% and pearson correlation coefficients exceeding 96%. (d–f) Histogram and distributional consistency comparisons between generated and ground truth physical parameters, respectively.

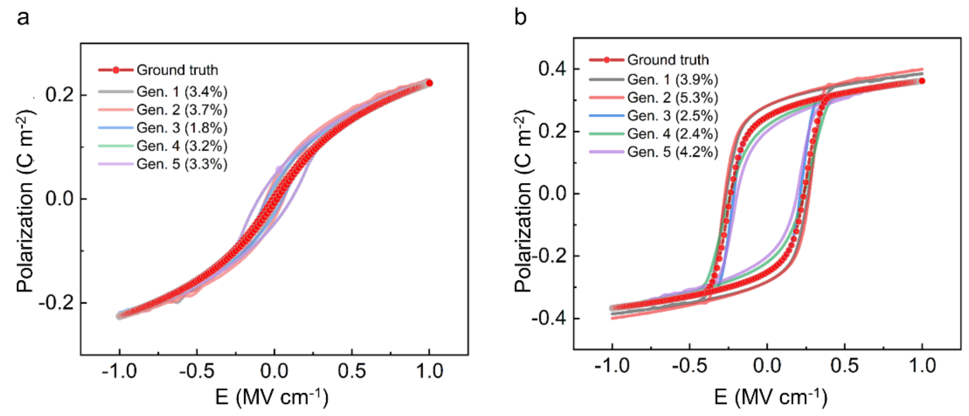

Supplementary Figure 6. Examples of microstructure generation evaluation with polarization profile conditions for two typical ferroelectric systems. (a) Simulated and generated *P*–*E* loops for a slim relaxor ferroelectric system. (b) Simulated and generated *P*–*E* loops for a conventional ferroelectric system. In both cases, the ground truth response (red) is compared with five independently generated microstructures (Gen. 1–5). The average NRMSE between generated and target curves is reported in parentheses.

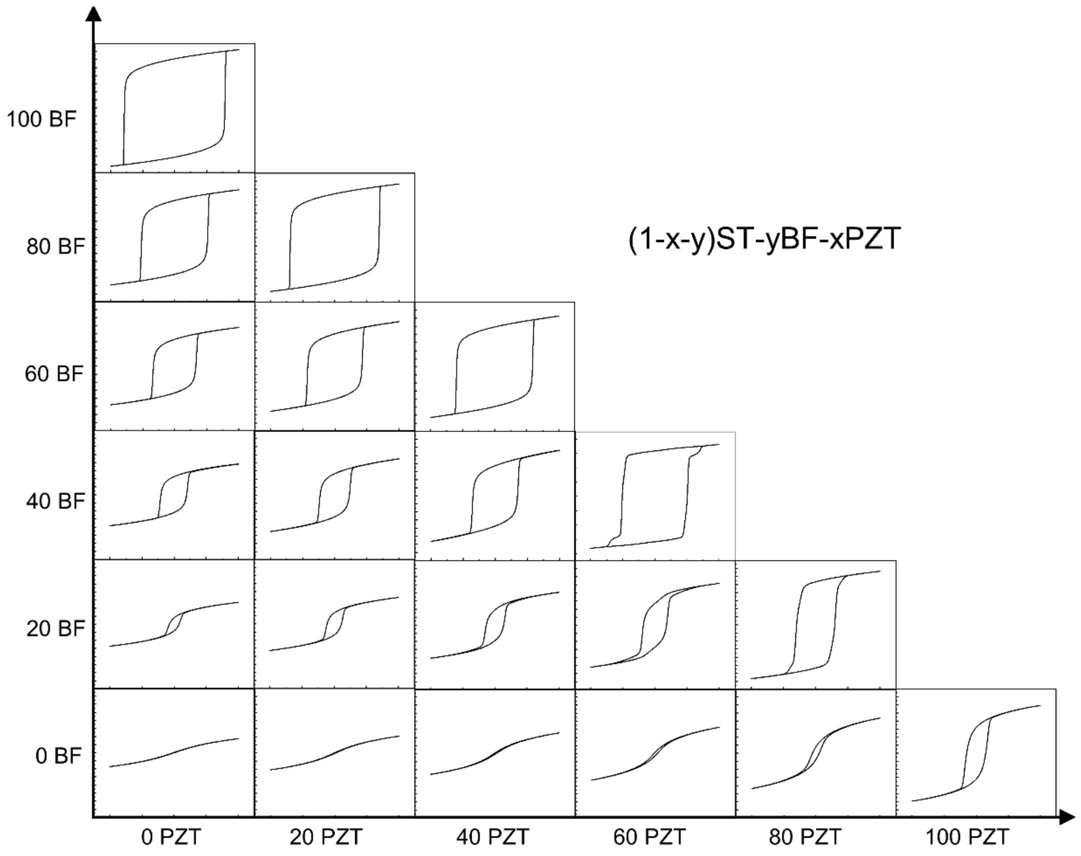

Supplementary Figure 7. *P-E* hysteresis loops of ST-BF-PZT multiphase solid solutions using phase-field simulation.

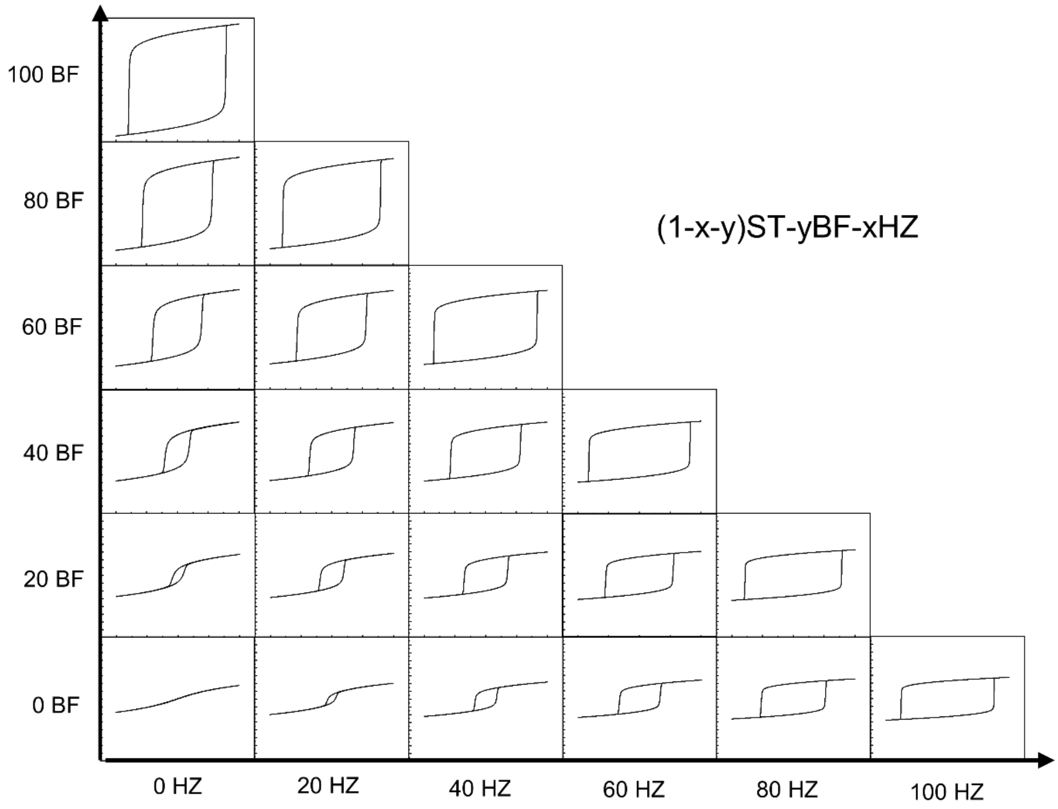

Supplementary Figure 8. *P-E* hysteresis loops of ST-BF-HZ multiphase solid solutions using phase-field simulation.

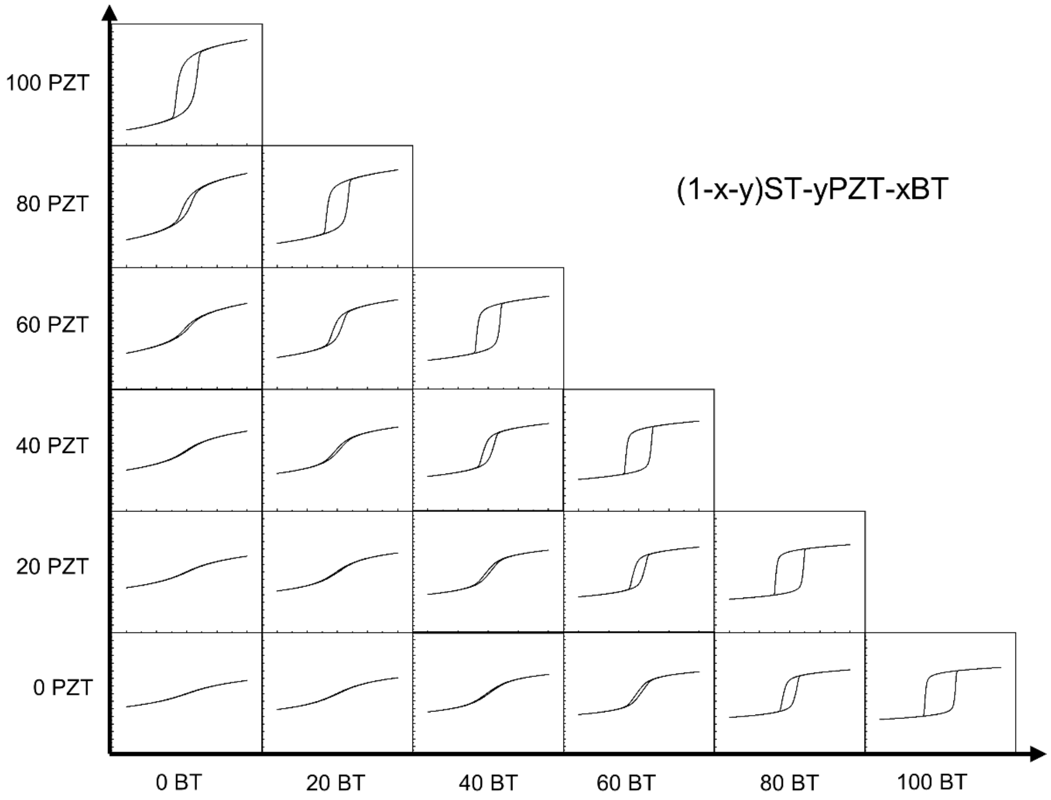

Supplementary Figure 9. *P-E* hysteresis loops of ST-PZT-BT multiphase solid solutions using phase-field simulation.

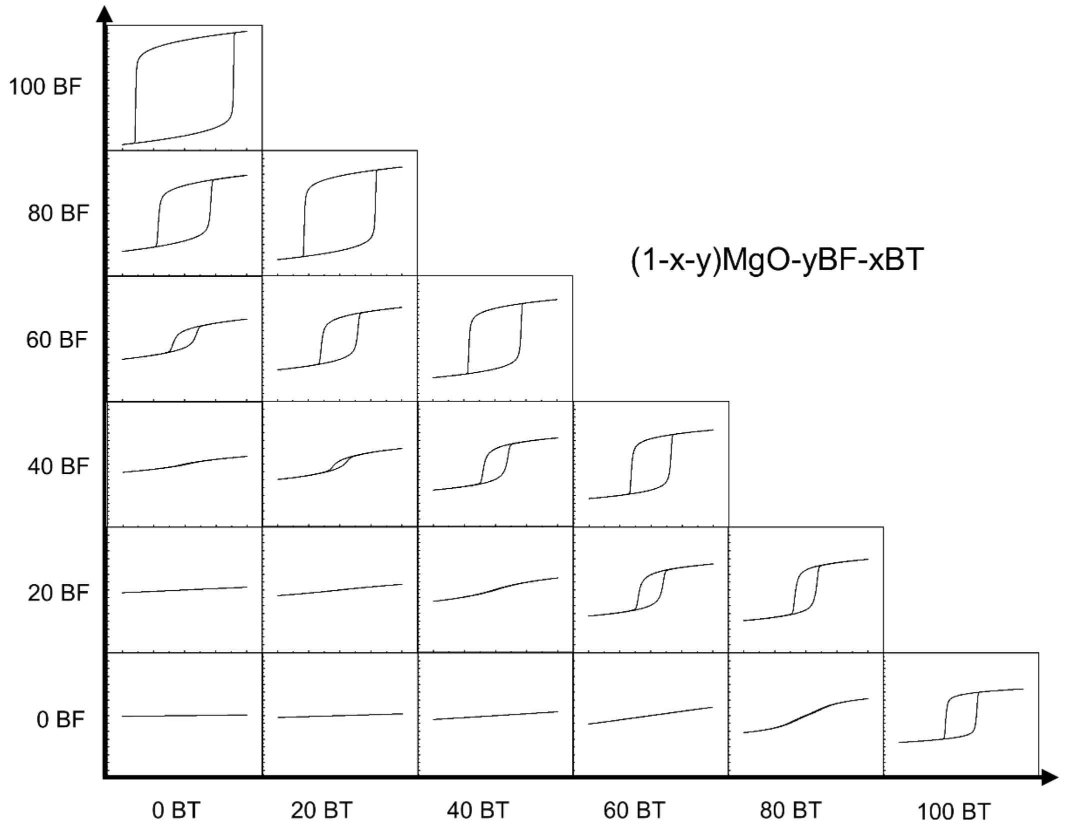

Supplementary Figure 10. *P-E* hysteresis loops of MgO-BF-BT multiphase solid solutions using phase–field simulation.

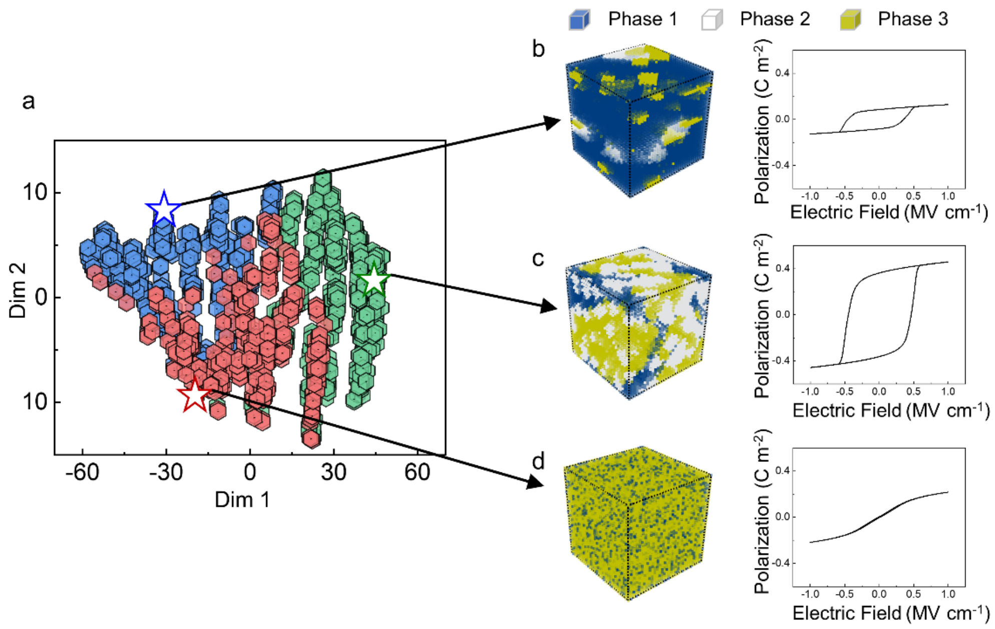

Supplementary Figure 11. Observed correlations between structural clusters and ferroelectric polarization behavior. (a) T-SNE visualization of polarization profiles for the MgO-BF-BT ternary system, with colors indicating structural clusters identified by k-means clustering. (b–d) Representative microstructures selected from each structural cluster and their corresponding *P*–*E* loops.

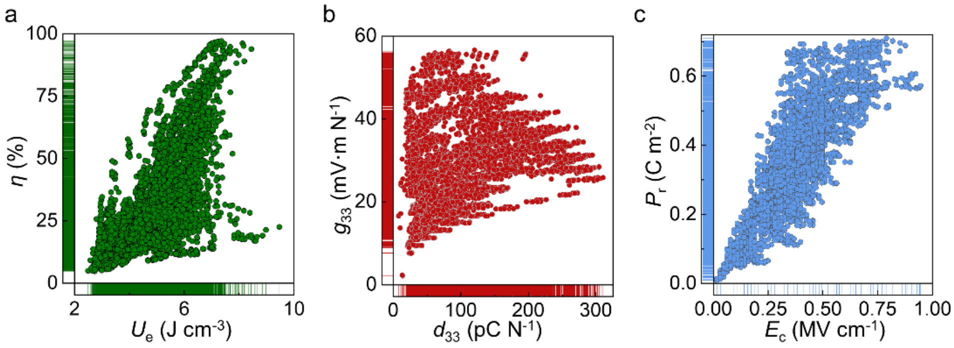

Supplementary Figure 12. Distributions of functional performance targets in capacitive energy storage, piezoelectric sensing and non-volatile memory scenarios. (a) Scatter plot of calculated recoverable energy density U_e versus efficiency η. (b) Scatter plot of simulated piezoelectric coefficient d_{33} versus dielectric permittivity ε. (c) Scatter plot of coercive field E_c versus remanent polarization P_r, highlighting the trend between switching sharpness and field stability. Marginal histograms show the distribution density along each axis

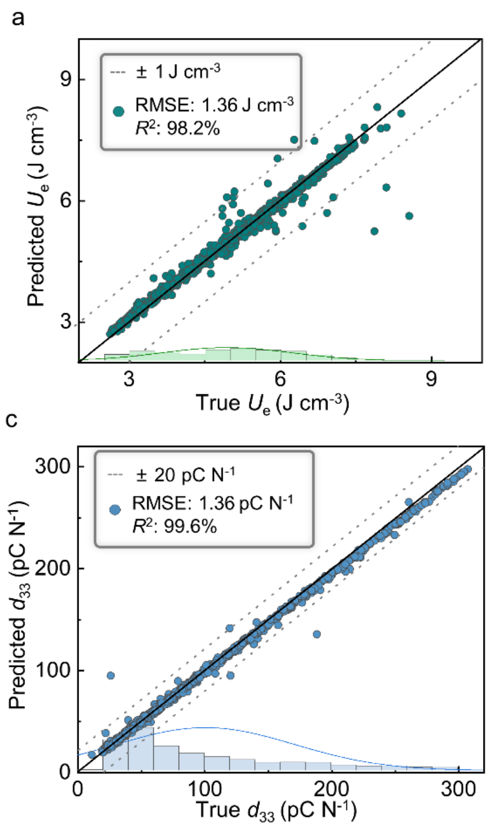

Supplementary Figure 13. Performance evaluation of the predictor in capacitive energy storage and piezoelectric sensing applications in test set. Scatter plots and histograms comparing predicted versus true values for key functional targets, including (a) U_e; (b) η; (c) d_{33}; (d) g_{33}. The x-axes denote ground truth values, and the y-axes indicate model predictions. The performance evaluation of predictors of P_r and E_c can be found in Fig. 2e–f.

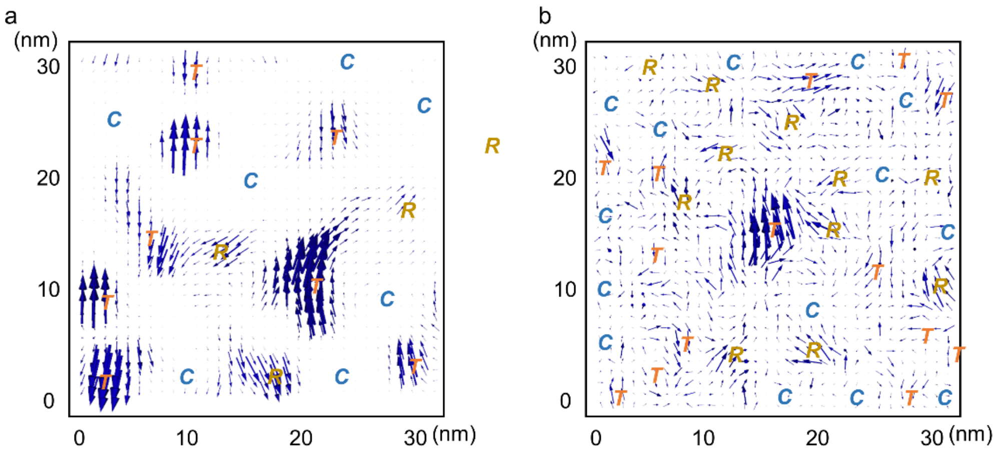

Supplementary Figure 14. 2D domain structure corresponds to Fig. 5a. (a) Domain structure corresponding to the optimal microstructure in the dataset. (b) The domain structure corresponding to the optimal microstructure was generated by DeepFerro.

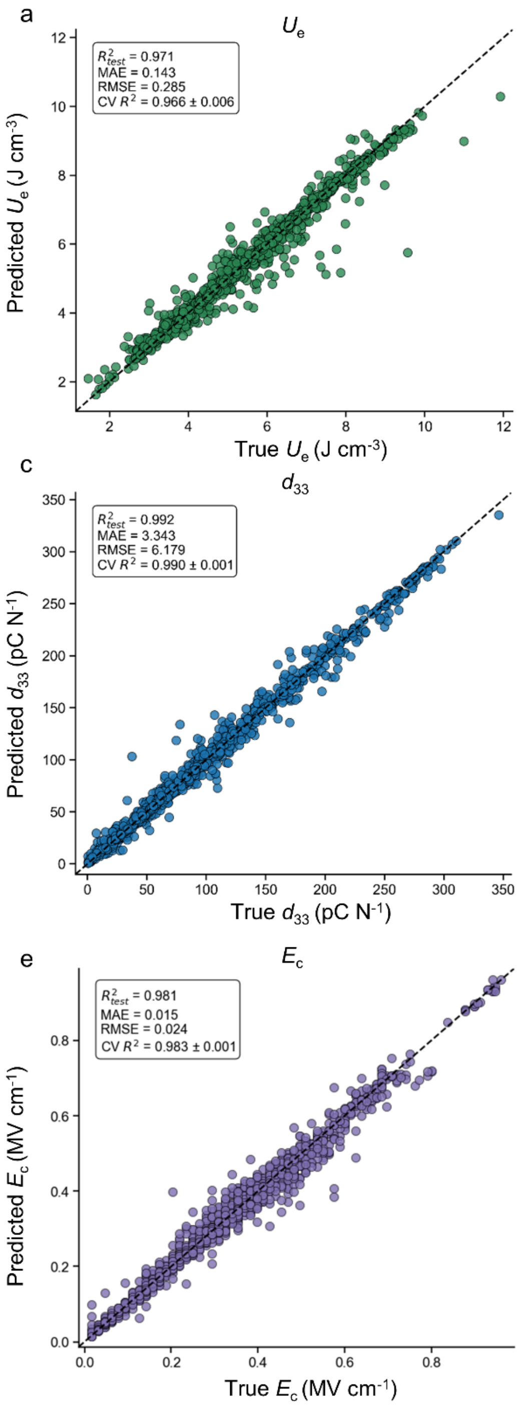

Supplementary Figure 15. Predictive performance of the random forest models used for feature importance analysis including (a) U_e; (b) η; (c) d_{33}; (d) g_{33}; (e) E_c; (f) P_r.

Supplementary Tables

Supplementary Table 1. The effect of Guidance scale λ on the generation performance.

λ	MSE	MAE	R^2	Person Correlation
**1**	0.0703	0.1822	93.52%	0.9697
**3**	0.0497	0.1497	95.44%	0.9777
**5**	0.0630	0.1618	94.22%	0.9718
**10**	0.0878	0.1962	91.87%	0.9618

Supplementary Table 2. Comparison of methods, software and running speeds for different tasks in materials microstructure design. The predictor was composed of 25.285 million parameters, whereas the generator consisted of a VAE (246,139 million) and a U-Net (488,753 million), yielding a total of 734,892 million parameters. All models are executed on an Intel Xeon Gold 5218 processor, featuring 16 cores and 32 threads, with a base clock speed of 2.30 GHz and supporting Turbo Boost frequencies up to 3.90 GHz, thereby providing substantial parallel processing power. The system is equipped with 503 GB of physical memory, which enables the efficient handling of large-scale data processing and complex computational tasks. Training of machine learning models is dependent on the utilization of eight NVIDIA RTX 3090 GPUs, each of which is equipped with 24 GB of VRAM. Each GPU is assigned six physical CPU cores and 60 GB of memory, resulting in a GPU: CPU: memory ratio of 1:6:60. Training utilizes CUDA 11.7, ensuring optimal parallel computation and acceleration support.

Task	description	Runtime
**Experimental design (1 structure)**	Theoretical guidance and empirical trial	2 week
**Phase field (1 structure)**	CPU training of numerical simulation	20min (32*32*32), 2 h (100*100*100) (1 core)
**Dataset construction**	CPU training of numerical simulation	5.4 days (16 core)
**DeepFerro training**	GPU training based on Pytorch	5 days
**DeepFerro generation (1 structure)**	GPU training based on Pytorch	10 seconds

Supplementary Table 3. Definitions of the constituent, content and microstructural descriptors forming the hierarchical design space.

Category	Descriptor	Symbol	Definition
**Constituent**	Landau coefficients	$a_1, ...a_{123}$; $m_1, ...m_{123}$; $w_1, ...w_{123}$	Sixth-order Landau coefficients of the ferroelectric phases.
	Permittivity	ε_i	Intrinsic dielectric permittivity of phase *i*.
	Maximum polarization	$P_{\max,i}$	Maximum polarization of the pure phase *i* under the applied field.
	Remanent polarization	$P_{\mathrm{r},i}$	Remanent polarization of the pure phase *i* after removing electric field.
	Coercive field	$E_{\mathrm{c},i}$	Coercive field of the pure phase *i*, defined from hysteresis loop.
**Content**	Phase fraction	φ_i	Volume fraction of phase *i*
**Morphology**	Largest connected cluster fraction	LCC_i	Volume fraction of phase *i* that belongs to the largest connected cluster of that phase.
	Mean equivalent radius	$r_{\mathrm{mean},i}$	Mean equivalent sphere radius of connected clusters of phase *i*.
	Cluster size polydispersity	$r_{\mathrm{cv},i}$	Coefficient of variation of the cluster radii of phase *i*.
	Morphological anisotropy ratio	R_{T}	Morphological anisotropy ratio.
**Alignment**	Mean contiguous thickness	$t_{\mathrm{mean},i}$	Mean thickness of contiguous segments of phase *i* along applied electric field direction.
	Median thickness along electric field	$t_{\mathrm{med},i}$	Median thickness of contiguous segments of phase *i* along applied electric field direction.
	90th-percentile thickness	$t_{\mathrm{p90},i}$	90th-percentile thickness of contiguous segments of phase *i* along applied electric field direction.
	Maximum contiguous thickness	$t_{\max,i}$	Maximum thickness of any contiguous segment of phase *i* along applied electric field direction.
	Principal structural angle	θ_{main}	Angle between the principal orientation of the composite

			microstructure and the applied electric field direction
**Interface**	Interfacial normal density along field	$\lambda_{\|\|}$	Density of phase boundaries
	Interfacial area fraction between phases	A_{ij}	Area of interfaces between phases *i* and *j*, normalized by the total interfacial area.
	Interfacial orientation fractions	$f_{ij}^{\|\|}, f_{ij}^{\angle}, f_{ij}^{\perp}$	Fractions of the *i*–*j* interfacial area

References

1. Chen X-X, Shen Z-H and Liu R-L *et al*. Programming polarity heterogeneity of energy storage dielectrics by bidirectional intelligent design. *Adv Mater* 2024; **36**: 2311721.

2. Resta R and Vanderbilt D. Theory of polarization: a modern approach. In: Rabe KM, Ahn CH and Triscone J-M (eds). *Physics of Ferroelectrics: A Modern Perspective*. Berlin: Springer, 2007, 31–68.

3. Xu K, Shi X and Shao C *et al*. Design of polar boundaries enhancing negative electrocaloric performance by antiferroelectric phase-field simulations. *npj Comput Mater* 2024; **10**: 150.

4. Sugathan S, Thekkepat K and Bandyopadhyay S *et al*. A phase field model combined with a genetic algorithm for polycrystalline hafnium zirconium oxide ferroelectrics. *Nanoscale* 2022; **14**: 14997–5009.

5. Liu D, Wang J and Wang J-S *et al*. Phase field simulation of misfit strain manipulating domain structure and ferroelectric properties in $PbZr_{(1-x)}Ti_xO_3$ thin films. *Acta Phys Sin* 2020; **69**: 127801.

6. Pan H, Li F and Liu Y *et al*. Ultrahigh–energy density lead-free dielectric films via polymorphic nanodomain design. *Science* 2019; **365**: 578-82.

7. Liu Y, Zhang Y and Xu Z *et al*. Ultrahigh capacitive energy storage through dendritic nanopolar design. *Science* 2025; **388**: 211-6.

8. Rombach R, Blattmann A and Lorenz D *et al*. High-resolution image synthesis with latent diffusion models. In: *Proceedings of the IEEE/CVF Conference on Computer Vision and Pattern Recognition*. Piscataway, NJ: IEEE Press, 2022, 10684-95.

9. Liu R-L, Wang J and Shen Z-H *et al*. AI for dielectric capacitors. *Energy Storage Mater* 2024; **71**: 103612.

10. Li F, Cabral MJ and Xu B et al. Ultrahigh piezoelectricity in ferroelectric ceramics by design. *Nat Mater* 2018; **17**: 349-54.

11. Scott JF and Paz de Araujo CA. Ferroelectric memories. *Science* 1989; **246**: 1400-

5.
